# Exploring the genome and transcriptome of the cave nectar bat *Eonycteris spelaea* with PacBio long-read sequencing

**DOI:** 10.1093/gigascience/giy116

**Published:** 2018-09-20

**Authors:** Ming Wen, Justin H J Ng, Feng Zhu, Yok Teng Chionh, Wan Ni Chia, Ian H Mendenhall, Benjamin PY-H Lee, Aaron T Irving, Lin-Fa Wang

**Affiliations:** 1 Programme in Emerging Infectious Diseases, Duke–NUS Medical School, 8 College Road, Singapore 169857, Singapore; 2 Conservation Division, National Parks Board, Singapore 259569, Singapore

**Keywords:** bat, *Eonycteris spelaea*, PacBio, Iso-Seq, genome assembly, alternative splicing

## Abstract

**Background:**

In the past two decades, bats have emerged as an important model system to study host-pathogen interactions. More recently, it has been shown that bats may also serve as a new and excellent model to study aging, inflammation, and cancer, among other important biological processes. The cave nectar bat or lesser dawn bat (*Eonycteris spelaea*) is known to be a reservoir for several viruses and intracellular bacteria. It is widely distributed throughout the tropics and subtropics from India to Southeast Asia and pollinates several plant species, including the culturally and economically important durian in the region. Here, we report the whole-genome and transcriptome sequencing, followed by subsequent *de novo* assembly, of the *E. spelaea* genome solely using the Pacific Biosciences (PacBio) long-read sequencing platform.

**Findings:**

The newly assembled *E. spelaea* genome is 1.97 Gb in length and consists of 4,470 sequences with a contig N50 of 8.0 Mb. Identified repeat elements covered 34.65% of the genome, and 20,640 unique protein-coding genes with 39,526 transcripts were annotated.

**Conclusions:**

We demonstrated that the PacBio long-read sequencing platform alone is sufficient to generate a comprehensive *de novo* assembled genome and transcriptome of an important bat species. These results will provide useful insights and act as a resource to expand our understanding of bat evolution, ecology, physiology, immunology, viral infection, and transmission dynamics.

## Data Description

### Background

Unique among the mammalian species as they are the only order with true powered-flight capability, bats have served as a unique model for studying evolutionary adaptation and morphological innovations, such as flight, echolocation, and longevity [1, 2]. More recently, bats have been increasingly recognized as an important reservoir harboring numerous pathogenic viruses while displaying minimal clinical signs of disease [3]. Indeed, comparing the genomes of bats with the genomes of other mammalian species has revealed an unexpected concentration of positively selected genes. These include those involved in DNA damage repair and innate immune functions, which may partially explain bats’ unique tolerance to deadly viruses and their unusually long life span [4]. Together, this highlights bats as an emerging model organism in the study of ecology, development, aging, and evolution.

Accurate assembly and annotation of genomes is a critical first step for further functional studies of genetic variation. To date, there are 14 draft bat genomes that are published and deposited in the National Center for Biotechnology Information (NCBI) (*Eidolon helvum, Eptesicus fuscus, Hipposideros armiger, Megaderma lyra, Miniopterus natalensis, Myotis brandtii, Myotis davidii, Myotis lucifugus, Pteropus alecto, Pteronotus parnellii, Pteropus vampyrus, Rhinolophus ferrumequinum, Rhinolophus sinicus, Rousettus aegyptiacus*; Supplementary Table S1) [2, 4–7]. Most of these genomes were assembled using only short Illumina sequencing reads (49–150 bp), with the exception of *R. aegyptiacus*, which utilized both short reads (data not released) from the Illumina HiSeq platform and long reads (data not released) from the Pacific Biosciences (PacBio) platform, resulting in a hybrid genome assembly. The long-read length of PacBio sequencing, which is available for both DNA and RNA sequencing (also known as isoform sequencing [Iso-Seq]), has shown considerable promise in genomics studies. For example, PacBio DNA sequencing has improved assembly of the human [8], gorilla [9], loblolly pine [10], and avian genomes [11], while Iso-Seq has helped deepen our understanding of alternative splicing in the chicken [12], coffee bean [13], and maize [14] transcriptomes.

**Figure 1:**
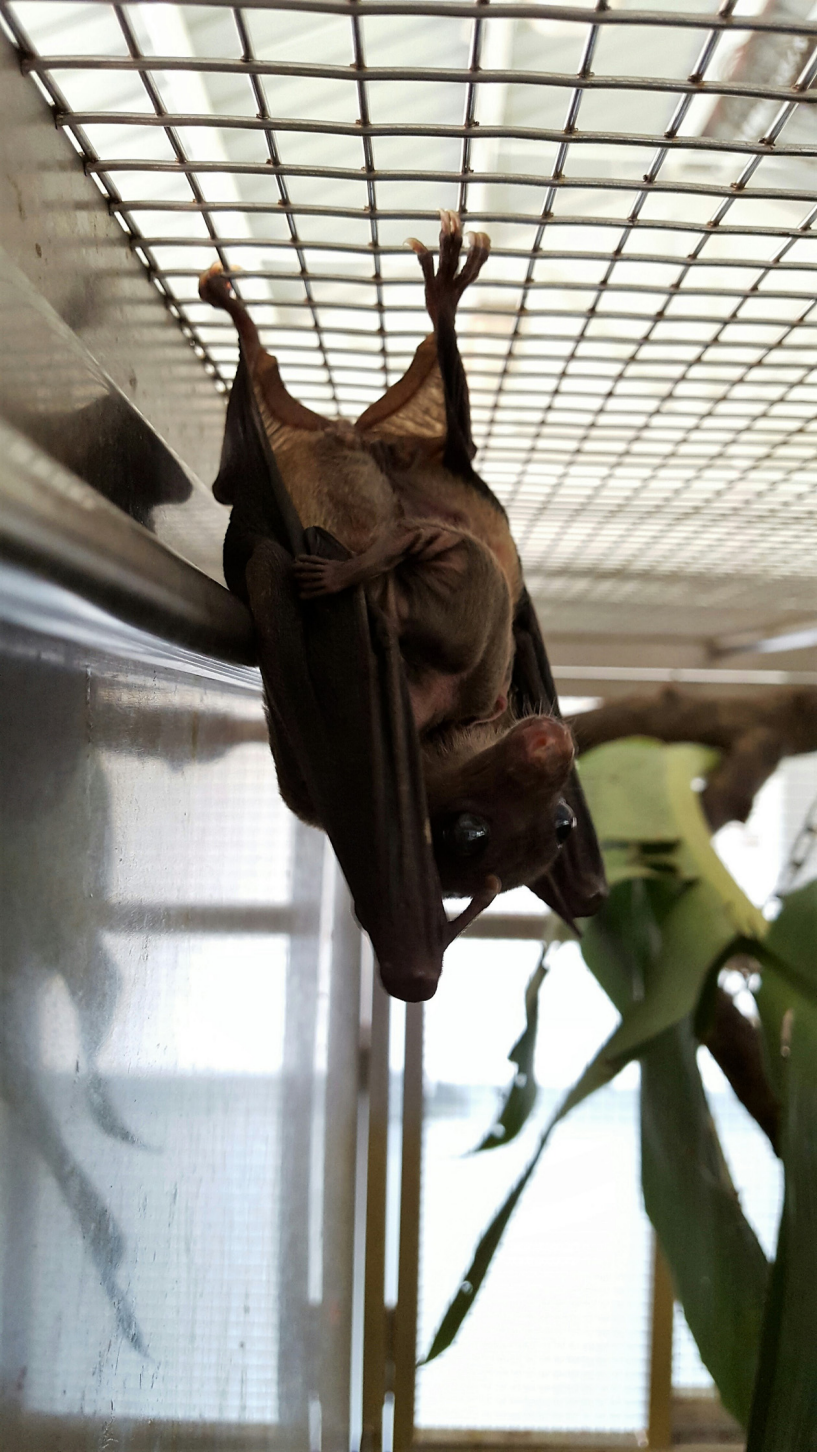
Female cave nectar bat (*Eonycteris spelaea*) with pup.

To produce a reliable genome resource and more thoroughly annotated genome than that of other bats, we employed PacBio technology to sequence both the genome and transcriptome of the cave nectar bat (also known as common nectar bat, dawn bat, common dawn bat, and lesser dawn bat [Fig. 1]), *Eonycteris spelaea* (*E. spelaea*, NCBI taxonomy ID: 58 065). We hoped the PacBio long-read technology would facilitate accuracy in annotating this evolutionary divergent species. *Eonycteris spelaea* is a specialist nectar-feeding bat that feeds predominantly on nectar and pollen. It is widely distributed over both the tropics and subtropics, throughout the Indomalayan region [15–17]. This species has been associated with pollination of durian and other fruits of both cultural and economic importance throughout Asia [18]. Additionally, this species has been identified as a carrier of orthoreoviruses, Lyssa virus, filoviruses, flavivirus, coronaviruses, and astroviruses [19–26]. The spread and abundance of this species make it an ideal subject for research purposes.

This newly assembled bat genome is 1.97 Gb in length, consisting of 4,470 sequences with a contig N50 of 8.0 Mbp. Identified repeat elements (REs) covered 34.65% of the genome, and 20,640 protein-coding genes were annotated. Also, 29,493 alternative spliceosomes for 10,607 genes were identified. Together, this resource and the identified regulatory elements provide information on the functional roles and relationships of various genomic loci, which in turn can be comparatively analyzed to further understand a vast array of bat-specific physiological features.

### Sampling and sequencing

For whole-genome sequencing, 175 single-molecule real-time (SMRT) cells were sequenced. This yielded 15,518,413 (∼161 Gb) reads with a mean subread length of 10,381 bp (standard deviation [SD], 7,424) and a N50 read length of 14,941 bp (Table 1). This translated into an ∼80x coverage for the target genome (∼2 Gb in length, estimates based on the average size of all previously sequenced 14 bat genomes, Supplementary Fig. S1). The ∼80x coverage is above the minimum 50x–60x coverage reported for self-correction of the high-error PacBio reads compared to Illumina reads [27].

**Table 1:** Data counts and library information for the *E. spelaea* genome

Library type	Insert size, kb	No. of subreads	N50 size	Total bp
DNA sequencing	20	15 518 413	14 941	161 109 271 053
Iso-Seq	1–2	107 230	1 409	148 990 235
	2–3	95 170	2 299	226 856 499
	3–6	104 687	3 700	403 584 865
	5–10	50 635	5 673	270 216 738
	Total	357 722	3 557	1 049 648 337

For Iso-Seq, 357,722 (∼1 Gb in total length) raw subreads (read length mean ± SD, 2934 ± 1775) were obtained (Table 1), representing an ∼20x coverage of the bat transcriptome repertoire (estimated using *P. alecto*’s 21,593 unique annotated genes, accounting for ∼0.05 Gb in length). After length-filtering and duplicate-collapsing (see Methods section), 31,639 unique full-length (FL) transcripts were used for subsequent analysis and gene annotation.

### Genome assembly and evaluation

After several rounds of parameter adjustments with the Falcon (v. 0.3.0) algorithm (see Methods section), we obtained a final 1.97-Gb assembly (named Espe.v1), which consists of 4,470 sequences with a contig N50 of 8.0 Mb (Table 2). We employed the Benchmarking Universal Single-Copy Orthologs (BUSCO) (v. 3) method [28] to evaluate the completeness of the genome annotation. The result showed that the vast majority (92.8%) of the representative mammal gene set (mammalia_odb9, which contains 4,104 single-copy genes that are highly conserved in mammals) was present in the assembled bat genome, demonstrating the completeness of gene set identification (Table 2). The Guanine-Cytosine (GC) content was 40.3%, similar to those of *P. alecto* (39.7%) and *R. aegyptiacus* (40.2%, Table 2). Overall, these metrics compare well with other recently published bat genomes, confirming Espe.v1 to be a reliable substrate for further genomic analyses.

**Table 2:** Comparison of genome features among *E. spelaea, P. alecto*, and *R. aegyptiacus*

Type	*E. spelaea*	*P. alecto*	*R. aegyptiacus*
Sequencing technology	PacBio	Illumina HiSeq	Illumina HiSeq + PacBio
Genome coverage	∼83x	∼110x	∼169x
Total genome length (bp)	1 966 861 576	1 985 975 446	1 910 250 568
Number of Contigs/scaffold	4 470/N.A.	170 164/65 598	3 049/2 490
Contigs/scaffold N50 (bp)	8 002 591/N.A.	31 841/15 954 802	1 488 988/2 007 187
GC level	40.3%	39.7%	40.02%
Repetitive elements^a^	34.65%	30.08%	29.58%
Number of coding genes	20,640	18,363	19,668
^b^(n = 4104)	C:92.8%,F:4.8%,M:2.4%	C:94.1%,F:3.7%,M:2.2%	C:93.5%,F:4.4%,M:2.1%

^a^Known mammalian repetitive elements deposit in Repbase (Update 20 160 829).

^b^BUSCO: Benchmarking Universal Single-Copy Orthologs; C: complete BUSCOs; F: fragmented BUSCOs; M: missing BUSCOs; N.A. not available.

### Genome annotation

RepeatMasker (v. 4.0.6) [29] was conducted with RMBlast (v. 2.2.28) to mask all the known mammalian transposon-derived REs. In order to compare the REs of *E. spelaea* with those of other genomes, we also performed the same analysis on all 14 available bat genomes on NCBI. Consistent with observations that PacBio technology is a better solution for solving repeats [8, 27], we found that known REs accounted for 34.65% of the genome in Espe.v1, which is the highest proportion among all bat genomes published to date (0.71% higher than that of the second RE-abundant bat genome, *R. sinicus*; Table 2, Supplementary Fig. S1). Similar to other bat genomes, the long interspersed nuclear elements (LINEs) and long terminal repeat elements constituted two of the highest proportion of all REs in Espe.v1, 51.32% and 18.05%, respectively (Supplementary Table S2).

**Figure 2:**
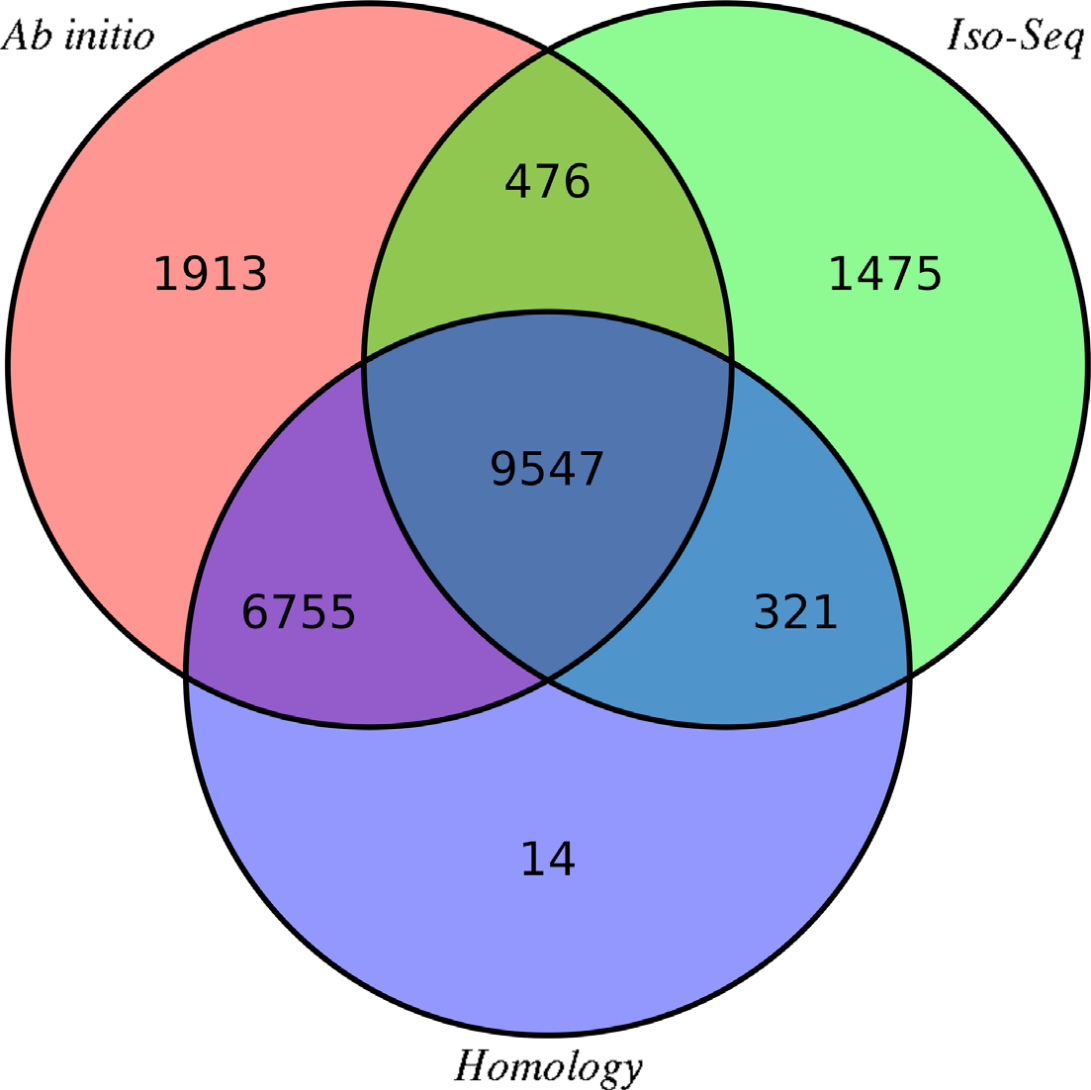
Venn diagram for coding gene predictions based on evidence sources. The different colors indicate various sources of evidence, and the values reflect the number of genes supported by each type of evidence.

After repeat masking, the genome was annotated with Maker2 (v. 2.31.9) [30] by integrating homologous prediction, *ab initio* prediction, and Iso-Seq-based prediction methods (see Methods section). As a result, the predicted gene set included 20,640 protein-coding genes (Fig. 2), of which 11,819 (57.2%) unique coding genes were supported by Iso-Seq and 16,637 (80.6%) were supported by homologous predictions. The relatively higher number of protein-coding genes predicted in *E. spelaea* compared to *P. alecto* and *R. aegyptiacus* is likely due to a more homologous approach being used to predict the gene models of *E. spelaea* than that of *P. alecto* and *R. aegyptiacus* (see Methods section). In summary, 18,588 (90.1%) of the coding genes were supported by at least one of the two types of prediction evidence (homologous and Iso-Seq evidence).

### Phylogenetic analysis

To evaluate the similarities and differences of available bat genomes and their evolutionary relationship, it is necessary to compare the phylogenies of *E. spelaea* to those of other bats and other mammalian species. Despite significant biological differences in the behaviors of bats, genetically some species are phylogenetically close, and this may impact any study of the homology between species. We identified single-copy orthologous gene clusters from 13 published genomes (8 bat genomes: *M. brandtii, M. lucifugus, M. davidii, E. spelaea, E. fuscus, P. alecto, P. vampyrus*, and *R. aegyptiacus* and 5 other mammalian genomes: *Homo sapiens, Mus musculus, Bos taurus*, and *Equus caballus* and *Monodelphis domestica*; with *M. domestica* as the outgroup; Supplementary Table S1) using the Proteinortho software [31]. In total, 3,185 single-copy gene families across all 13 species were identified.

**Figure 3:**
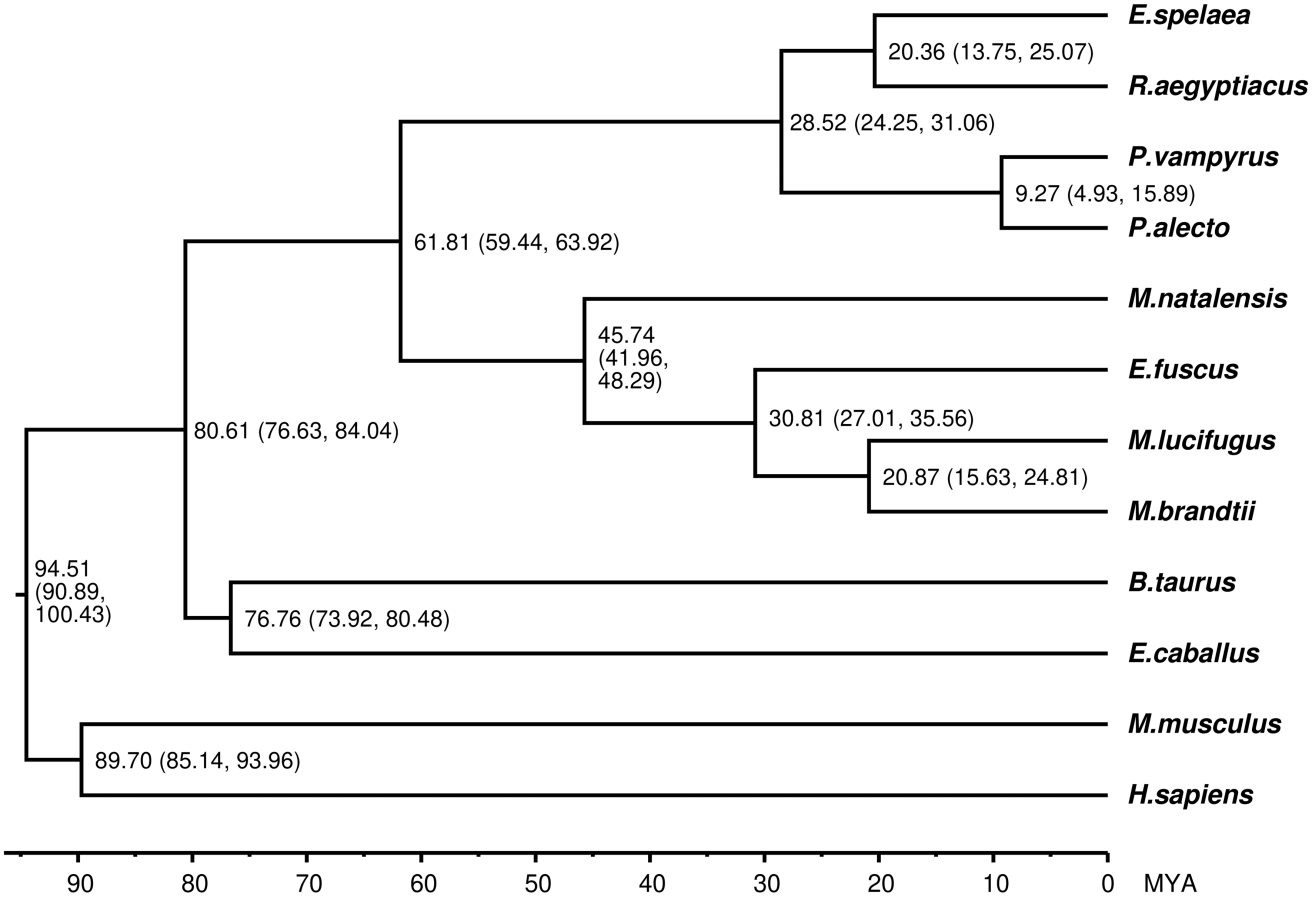
Maximum-likelihood phylogenetic analysis of 3,185 genes in bats and mammalian species. The estimated divergence time (100 Mya) is given at the nodes, with the 95% confidence intervals in parentheses. *Monodelphis domestica*, used as an outgroup species, was excluded in this figure.

The divergence times of *E. spelaea* and the 11 mammals (excluding *M. domestica*) were estimated using 999,609 4-fold degenerate sites from the 3,185 single-copy genes. The topological order and estimated divergence time of our phylogeny analysis (Fig. 3) are consistent with those from previous studies [4, 32], with bats, *E. caballus* (horse), and *B. taurus* (cow) clustering together within the Laurasiatheria superorder (bats diverging ∼80.61 million years ago [Mya) [4]. Our analysis also revealed that *E. spelaea* was the closest sister taxon to *R. aegyptiacus*, an Egyptian fruit bat that is distributed throughout Africa [33]. The divergence time between these two bat species was estimated at ∼20.36 Mya, indicating a relatively recent divergence. As *P. alecto, R. aegyptiacus*, and *E. spelaea* are all relatively close on the phylogenetic tree and all have reliable genome annotations, we focus on these three species for a more detailed comparison.

### Iso-Seq analysis

**Figure 4:**
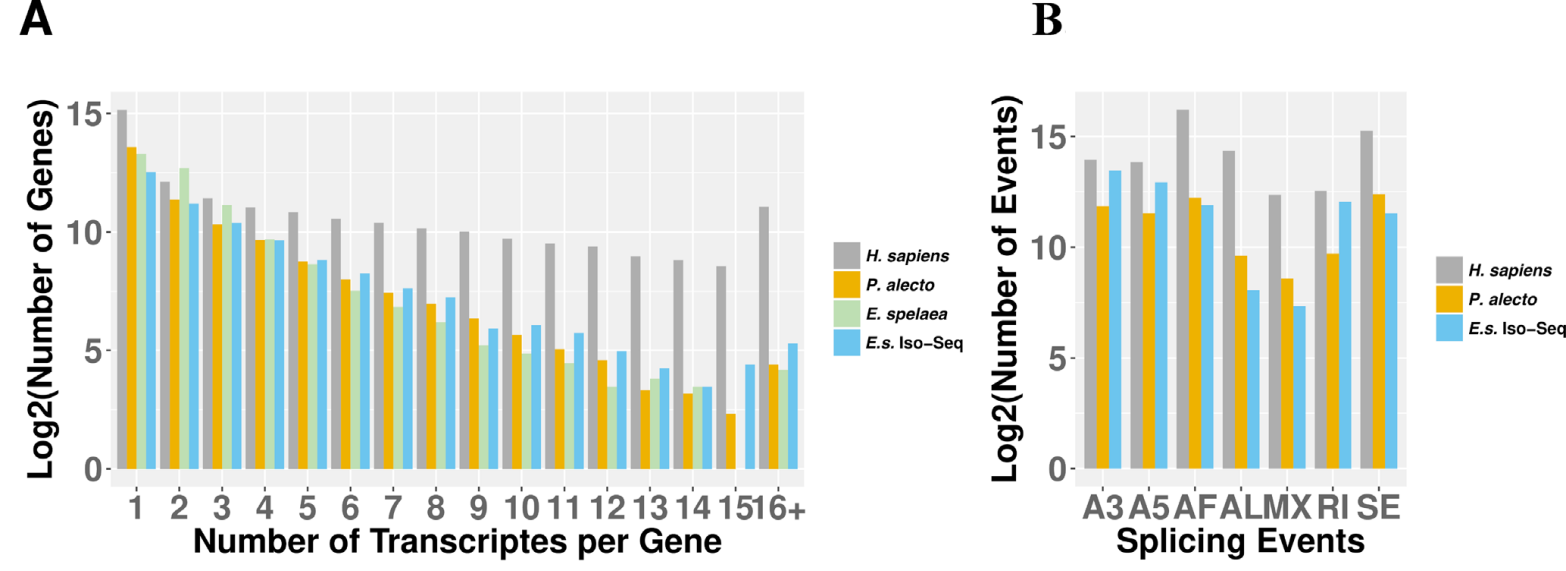
The alternative splicing of *E. spelaea*’s coding genes. **(A)** Comparison of number of alternative transcripts per annotated gene between *H. sapiens, P. alecto, E. spelaea*, and PacBio Iso-Seq *E. spelaea* transcriptomes. **(B)** Comparison of rate of occurrence for the different classes of alternative transcripts between *H. sapiens, P. alecto*, and the *E. spelaea* PacBio Iso-Seq transcriptome. Abbreviations: A5/A3: alternative 5′/3′ splice sites; AF/AL: alternative first/last exons; MX: mutually exclusive exons; RI: retained intron; SE: skipping exon.

A major advantage of Iso-Seq technology is that it captures FL gene isoforms without the need for any downstream assembly. The large number of unique transcripts recovered through Iso-Seq enabled us to make a general assessment of transcriptional complexity of the bat genome. Of the 31,639 FL Iso-Seq transcripts, 382 RE transcripts (98 LTRs, 31 DNA elements, 7 satellites, 244 LINEs, 1 short interspersed nuclear element, and 1 unknown RE) were filtered out from further analysis using RepeatMasker (see Methods section). The remaining 31,257 clean transcripts were compared against our homology and *ab initio* predicted genes. Of the 20,640 coding genes, we observed 10,033 (5,925 are supported by clean Iso-Seq transcripts) single transcript genes and 10,607 (5,894 are supported by clean Iso-Seq transcripts) alternatively spliced genes. Overall, we found an isoform-to-gene ratio of 1.92 (39,526 transcripts per 20,640 genes) in *E. spelaea*, which is lower than 3.62 (167,430 transcripts per 46,298 genes) in human, but higher than 1.49 (33,093 transcripts per 22,264 genes) in *P. alecto* (Fig. 4A). When narrowed down to genes only observed by Iso-Seq, we found an isoform-to-gene ratio of 2.39 (28,289 transcripts per 11,819 genes), suggesting that PacBio Iso-Seq technology has significantly increased the isoform diversity discovery of *E. spelaea*’s transcriptome compared to that of *P. alecto*’s transcriptome, which was sequenced using the Illumina RNA-Seq technology (Fisher exact test, *P* value < 10^−5^).

The alternative transcript events were further classified into skipping exon, alternative 5′/3′ splice sites, mutually exclusive exons, retained intron, and alternative first/last exons by SUPPA software (last updated 02/07/2017) [34]. We identified 30,487 alternative splicing events in the Iso-Seq dataset, which is 5.80-fold lower than that in human but 1.62-fold higher than that in *P. alecto* (Fig. 4B). In particular, alternative 5’- (7,783 events) and 3’- (11,258 events) splicing were two of the most predominant events in the *E. spelaea* spliceosome repertoire (Fig. 4B). Our results provide the first comprehensive overview of splice variants in any bat species using a direct sequencing analysis approach rather than *in silico* analysis.

### Conclusions and discussion

In this study, we provided the first assembly of a bat genome solely using PacBio long-read sequencing technology. The *E. spelaea* genome assembly exemplifies the power of long-read sequencing technologies in rapid *de novo* assembly of a nonmodel genome and alternative-splicing isoform identification. Compared to the hybrid assembled *R. aegyptiacus* genome, the “PacBio-only” assembly was faster, more straightforward, and possessed higher contiguous N50 contigs and better repeat-resolving with a minimum difference (0.7%) in gene integrity. Thus, our study provides a high-quality reference genome for use in any future comparative studies. Even without scaffolding, these highly contiguous contigs and FL gene transcripts will be helpful to researchers to extract more accurate genomic loci information of their genes of interest, saving a great amount of energy, resources, and time. Our Iso-Seq results have increased our understanding of the complexity of the bat transcriptome and aided in alternative transcript identification. This complexity in the transcriptome of bats is still likely to be underestimated since Iso-Seq was performed at a relatively shallow depth (∼20x coverage) in this study. We would like to further highlight that this complexity is attributed by the type and number of alternative transcription events, as well as previously unannotated transcripts in bats. As more and more transcriptome data become available for *E. spelaea*, e.g., Illumina transcriptome sequencing of various tissues, this will aid in the identification of other alternative transcripts and continuously improve the annotation of the genome and the accuracy of protein coding sequences, as has been evident with the *P. alecto* genome since its first release. Taken together, we have provided a valuable resource, an *E. spelaea* genome and transcriptome database, for future comparative and functional studies, as well as demonstrated the advantages of employing the latest long-read sequencing technology in such studies of exotic species.

## Methods

### Bat sample processing

*Eonycteris spelaea* was captured in Singapore at dusk using mist nets and transferred to clean customized bat bags for transportation. All animal processing work was conducted in accordance with approved guidelines and methods in line with permits obtained from the National Parks Board, Singapore (NP/RP14–109) and animal ethics approval from the National University of Singapore (B16–0159). Bats were euthanized using isoflurane and exsanguinated via cardiac bleed. Various tissue samples, as detailed in Supplementary Table S3, were harvested and preserved in RNA*later* stabilization solution (Invitrogen). Tissues were homogenized, and RNA was extracted using the RNeasy Mini Kit (Qiagen) with an additional on-column DNase digestion step using RNase-Free DNase Set (Qiagen). Extracted RNA was subsequently eluted in RNase-Free water and stored at –80 °C.

For genomic DNA extraction, fresh lung and kidney samples were snap frozen in liquid nitrogen immediately upon harvesting and pounded into powder form before extraction using the Gentra Puregene Tissue Kit (Qiagen).

### DNA and Iso-Seq sequencing

Genomic DNA was extracted from a single male *E. spelaea*. Two DNA libraries, derived from kidney and lung samples, were constructed using SMRTbell Template Prep Kits 1.0 (Pacific Biosciences) with a 20 kb insert size. SMRT sequence data were generated using P6v2 polymerase binding and C4 chemistry (P6-C4) kits over a 6-hour movie run-time on the PacBio RSII instrument. Library construction and sequencing runs were performed by a commercial sequencing provider DNA Link Inc. (Korea).

For Iso-Seq sequencing, total RNA from a panel of tissues (Supplementary Table S3) was extracted from two individuals, a female and a male. Tissue RNA from individual bats was then pooled together. Four libraries of 1–2 kb, 2–3 kb, 3–6 kb, and 5–10 kb insert sizes were generated using the P6-C4 kits and subsequently sequenced. In total, 34 SMRT cells were sequenced on the PacBio RS II platform over a 3- to 4-hour movie run time. Library construction and Iso-Seq runs were performed at the Duke-NUS Genome Biology Facility (Duke-NUS Medical School, Singapore).

### Genome assembly

PacBio subreads were filtered with default parameters and submitted to Falcon ([35], v. 0.3.0) for genome assembly. For the final assembly (Espe.v1), 15,518,413 subreads (read length mean ± SD, 10,382 ± 7,424) were used for assembly, with a length-cutoff parameter of 2 kb for initial mapping to build pre-assembled reads (also referred to as error-corrected preads). The pre-assembly module is a built-in module in Falcon for error-correcting PacBio subreads. A total of 15,470,844 preads were generated (read length mean ± SD, 7,034 ± 5,874) from the PacBio subreads. Preads over 10 kb were used (length-cutoff-pr) to seed pre-assembly. Daligner was used to detect all pairwise local overlapping regions between subreads and also between corrected preads. Overlapping options were set to “-v -B128 -M40 -e.70 -l2000 -s400” for pre-assembly and “-v -B128 -M40 -h45 -e.96 -l500 -s400” for alignment of corrected preads. To reduce computation and assembly graph complexity, overlaps built by corrected preads were filtered by “–max-diff 300 –max-cov 400 –min-cov 2 –bestn 20” to remove the transitive reducible overlaps. Consensus was built from the overlapping preads on “–output-multi –min-idt 0.70 –min-cov –max-n-read 400.” Finally, primary and associated contigs were polished using Quiver with default parameters.

### Iso-Seq analysis

For Iso-Seq analysis, raw reads were classified into circular consensus sequences (CCS) and non-CCS subreads by ToFu (v. 4.1) [36], and FL CCS reads were filtered out if both the 5′- and 3′-cDNA primers were present, as well as a polyA tail signal preceding the 3′-primer. To improve consensus accuracy, the isoform-level clustering algorithm (iterative clustering for error correction) and Quiver were applied to generated FL transcripts with ≥99% post-correction accuracy. Next, the Quiver-polished FL CCS reads were mapped to the assembled genome using GMAP (v. 2018–01-26) [37] and collapsed by the pbtranscript-ToFU package ([38], last updated: 10/15/2015) with default parameters to collapse redundant transcripts. Collapsed transcripts were screened for REs by RepeatMasker (v. open-4.0.6) [39] to mask all mammalian RE sequences. Transcripts with ≥70% bases masked were denoted as REs and discarded from further analysis. Alternative splicing events in the repeat-cleaned Iso-Seq reads, human (Ensembl GRCh38.p10), and *P. alecto* (NCBI assembly ASM32557v1) mRNAs were classified with SUPPA (last updated 02/07/2017) under default parameters.

### Genome annotation

Maker2 (v. 2.31.9) [30] was utilized to perform genome annotation. Repetitive genomic elements were identified and masked from annotation with RepeatMasker using the Repbase database (Update 20 160 829) [40]. Cleaned Iso-Seq transcripts (see above) were used as transcript evidence. Augustus (v. 2.7) [41] and SNAP (release 11/29/2013) [42] were used as *ab initio* gene predictors. Unique protein sequences from eight different mammals (*B. taurus, Canis familiaris, E. caballus, H. sapiens, M. musculus, M. lucifugus, P. alecto, P. vampyrus*; Supplementary Table S1) were downloaded from Ensembl (last accessed: 5/15/2017) [43] and used for homology-based prediction. The Maker2 pipeline was first run on the masked genome using the Iso-Seq transcriptome to infer gene predictions (est2genome  =  1), and training files for the *ab initio* gene predictors Augustus and SNAP were generated based on these results. Then, the annotation pipeline was run iteratively two additional times using the Iso-Seq transcriptome as evidence (est2genome  =  0) and providing new training files with each run. At this point, the protein-homology evidence was set to include all unique proteins in the eight different mammals. Next, Maker predict transcripts were merged with Iso-Seq transcripts and collapsed using pbtranscript-ToFU to include all the unique alternative spliced transcripts. Finally, low-quality genes shorter than 50 amino acids and/or exhibiting premature termination were removed to produce the final gene set.

### Phylogenetic analysis

Phylogenetic tree construction and divergence time estimation were performed as described in [4]. Briefly, Proteinortho software (v. 5.16b) [31] was used to identify the single-copy orthologous genes under default parameter setting. Using *M. domestica* as an outgroup, we identified 3,185 single-copy orthologous genes from *E. spelaea* and 11 other mammalian genomes (as described above). The coding sequence from each single-copy family was aligned by MUSCLE (v. 3.8.31) [44]. Four-fold degenerate sites were extracted from each gene alignment by an in-house Python script and concatenated to one super gene for each species. Then, RAxML (v. 8.2.11) [45] was applied to build phylogenetic trees for the concatenated sequences as described [4]. A total of 1,000 bootstrap replicates were employed to assess branch reliability in RAxML. Last, PAML (v. 4.9c) mcmctree [46] was used to determine split times based on the topology obtained in the RAxML analysis [4]. The gamma prior for the overall substitution rate was described by shape and scale parameters that were set as 1 and 11.1, respectively, calculated according to the substitution rate per time unit using PAML baseml [47]. Fossil calibrations were retrieved from the TimeTree database (last accessed 12/15/2017) [48]. Other parameters were set as default. The PAML mcmctree pipeline was run two independent times to confirm convergence and all acceptance proportions fall in the interval (20%, 40%).

## Availability of supporting data

Genome data are available in the NCBI database ( project accession PRJNA427241). Further supporting data can be found in the *GigaScience* repository, GigaDB [49].

## Additional files

**Figure S1:** Scatter plot of the genome size and proportion of repeat content for 15 bats genomes.

**Table S1:** Reference genomes used in this study.

**Table S2:** Descriptive statistics of the *E. spelaea* genome repeat elements using RepeatMasker. RepBase Update 20 160 829.

**Table S3:** Detailed listing of *E. spelaea* tissue RNA used for Iso-Seq analysis

## Abbreviations

BUSCO: Benchmarking Universal Single-Copy Orthologs; CCS: circular consensus sequence; FL: full-length; GC: Guanine-Cytosine; Iso-Seq: isoform sequencing; LINE: long interspersed nuclear element; Mya: million years ago; NCBI: National Center for Biotechnology Information; P6-C4: P6v2 polymerase binding and C4 chemistry; PacBio: Pacific Biosciences; RE: repeat element; SD: standard deviation; SMRT: single-molecule real-time.

## Competing interests

The authors declare that they have no competing interests.

## Funding

This work was funded by the Singapore National Research Foundation Competitive Research Programme (grant NRF2012NRF-CRP001–056). A.T.I. is supported by a New Investigator's Grant from the National Medical Research Council of Singapore (NMRC/BNIG/2040/2015). I.H.M. was supported by a New Investigator's Grant from the National Medical Research Council of Singapore (NMRC/BNIG/2005/2013). B.P.Y-H.L. was supported by a research grant from the Wildlife Reserves Singapore Conservation Fund (WRSCF).

## Author contributions

L-F.W. and J.H.J.N. conceived and designed the study; I.H.M., B.P.Y-H.L., C.Y.T., A.T.I and J.H.J.N. led the bat field work; C.Y.T. and J.H.J.N. performed the experimental processing of samples; M.W. and F.Z. led the sequence analysis; and F.Z. and A.T.I. contributed to data analysis. All authors contributed to manuscript writing, reviewing, and approval of the final version for submission.

## Supplementary Material

GIGA-D-18-00099_(Original_Submission).pdf

GIGA-D-18-00099_Revision_1.pdf

GIGA-D-18-00099_Revision_2.pdf

giy116_Response_to_Reviewer_Comments_Report_(Original_Submission).pdf

Response_to_Reviewer_Comments_Report_Revision_1.pdf

Reviewer_3_Original_Submission_(Attachment).pdf

Reviewer_1_Report_(Original_Submission) -- Michael Hiller 4/20/2018 Reviewed

Reviewer_2_Report_(Original_Submission) -- Xiuguang Mao 4/24/2018 Reviewed

Reviewer_3_Report_(Original_Submission) -- Graham Hughes 5/1/2018 Reviewed

Reviewer_1_Report_Revision_1 -- Michael Hiller 4/20/2018 Reviewed

Reviewer_2_Report_Revision_1 -- Xiuguang Mao 8/3/2018 Reviewed

Reviewer_3_Report_Revision_1 -- Graham Hughes 8/5/2018 Reviewed

Supplement File
