# Supplementary material for: Exploring the genome and transcriptome of the cave nectar bat Eonycteris spelaea with PacBio long-read sequencing
Source: Gigascience. 2018 Sep 20;7(10):giy116. doi: 10.1093/gigascience/giy116 (PMC6177735; doi:10.1093/gigascience/giy116)
Supplement: GIGA-D-18-00099_Revision_1.pdf [file giy116_giga-d-18-00099_revision_1.pdf]

GigaScience

Exploring the genome and transcriptome of the cave nectar bat Eonycteris spelaea with PacBio long-read sequencing

--Manuscript Draft--

**Manuscript Number:**	GIGA-D-18-00099R1
**Full Title:**	Exploring the genome and transcriptome of the cave nectar bat Eonycteris spelaea with PacBio long-read sequencing
**Article Type:**	Data Note
**Funding Information:**	National Research Foundation Singapore (NRF2012NRF-CRP001-056) — Prof Lin-Fa Wang<br>National Medical Research Council (NMRC/BNIG/2040/2015) — Dr Aaron Irving<br>Wildlife Reserves Singapore Conservation Fund (SG) — Dr Benjamin Lee

Abstract:

Background
In the past two decades, bats have emerged as an important model system to study host-pathogen interactions. More recently, it has been shown that bats may also serve as a new and excellent model to study aging, inflammation and cancer among other important biological processes. The cave nectar bat or lesser dawn bat (Eonycteris spelaea), is known to be a reservoir for several viruses and intracellular bacteria. It is a widely-distributed bat species throughout the tropics and subtropics from India to Southeast Asia, and pollinates several plant species, including the culturally and economically important durian in the region. Here, we report the whole-genome and transcriptome sequencing, followed by subsequent de novo assembly of the E. spelaea genome solely using the PacBio® long-read sequencing platform.

Findings
The newly assembled E. spelaea genome is 1.97 Gb in length and consists of 4,470 sequences with a contig N50 of 8.0 Mb. Identified repeat elements covered 34.65% of the genome and 20,640 unique protein coding genes with 39,526 transcripts were annotated.

Conclusions
We demonstrated that PacBio® long-read sequencing platform alone is sufficient to generate a comprehensive de novo assembled genome and transcriptome of an important bat species. These results will provide useful insights and act as a resource to expand our understanding of bat evolution, ecology, physiology, immunology, viral infection and transmission dynamics.

**Corresponding Author:**	Lin-Fa Wang<br>Duke-NUS Medical School<br>Singapore, SINGAPORE
**Corresponding Author Secondary Information:**	
**Corresponding Author's Institution:**	Duke-NUS Medical School
**Corresponding Author's Secondary Institution:**	
**First Author:**	Lin-Fa Wang
**First Author Secondary Information:**	
**Order of Authors:**	Lin-Fa Wang
	Ming Wen
	Justin Ng
	Yoke Teng Chionh

Powered by Editorial Manager® and ProduXion Manager® from Aries Systems Corporation

	Wan Ni Chia
	Ian Mendenhall
	Benjamin Lee
	Aaron Irving
	Feng Zhu
**Order of Authors Secondary Information:**	
**Response to Reviewers:**	List of responses attached in the cover letter
**Additional Information:**	
**Question**	**Response**
Are you submitting this manuscript to a special series or article collection?	No
**Experimental design and statistics**<br><br>Full details of the experimental design and statistical methods used should be given in the Methods section, as detailed in our Minimum Standards Reporting Checklist. Information essential to interpreting the data presented should be made available in the figure legends.<br><br>Have you included all the information requested in your manuscript?	Yes
**Resources**<br><br>A description of all resources used, including antibodies, cell lines, animals and software tools, with enough information to allow them to be uniquely identified, should be included in the Methods section. Authors are strongly encouraged to cite Research Resource Identifiers (RRIDs) for antibodies, model organisms and tools, where possible.<br><br>Have you included the information requested as detailed in our Minimum Standards Reporting Checklist?	Yes
**Availability of data and materials**<br><br>All datasets and code on which the	Yes

conclusions of the paper rely must be either included in your submission or deposited in publicly available repositories (where available and ethically appropriate), referencing such data using a unique identifier in the references and in the “Availability of Data and Materials” section of your manuscript.<br><br>Have you have met the above requirement as detailed in our Minimum Standards Reporting Checklist?	

Manuscript

Click here to access/download;Manuscript;GigaSci-Es genome-Text-Submission 180725.docx

Click here to view linked References

Exploring the genome and transcriptome of the cave nectar bat *Eonycteris spelaea* with PacBio long-read sequencing

Ming Wen[1,†], Justin H. J. Ng[1,†], Feng Zhu[1], Chionh Yok Teng[1], Wan Ni Chia[1], Ian H. Mendenhall[1], Benjamin P. Y-H. Lee[2], Aaron T. Irving[1,], Lin-Fa Wang[1,*]*

[1]Programme in Emerging Infectious Diseases, Duke–National University of Singapore Medical School, Singapore 169857, Singapore

[2]Conservation Division, National Parks Board, Singapore 259569, Singapore

[†]Equal contribution

* Corresponding authors: Aaron Irving, email: aaron.irving@duke-nus.edu.sg; Lin-Fa Wang, email: linfa.wang@duke-nus.edu.sg

ABSTRACT

Background

In the past two decades, bats have emerged as an important model system to study host-pathogen interactions. More recently, it has been shown that bats may also serve as a new and excellent model to study aging, inflammation and cancer among other important biological processes. The cave nectar bat or lesser dawn bat (*Eonycteris spelaea*), is known to be a reservoir for several viruses and intracellular bacteria. It is a widely-distributed bat species throughout the tropics and subtropics from India to Southeast Asia, and pollinates several plant species, including the culturally and economically important durian in the region. Here, we report the whole-genome and transcriptome sequencing, followed by subsequent *de novo* assembly of the *E. spelaea* genome solely using the PacBio® long-read sequencing platform.

Findings

The newly assembled *E. spelaea* genome is 1.97 Gb in length and consists of 4,470 sequences with a contig N50 of 8.0 Mb. Identified repeat elements covered 34.65% of the genome and 20,640 unique protein coding genes with 39,526 transcripts were annotated.

Conclusions

We demonstrated that PacBio® long-read sequencing platform alone is sufficient to generate a comprehensive *de novo* assembled genome and transcriptome of an

important bat species. These results will provide useful insights and act as a resource to expand our understanding of bat evolution, ecology, physiology, immunology, viral infection and transmission dynamics.

Keywords

Bat, *Eonycteris spelaea*, PacBio, Iso-Seq, genome assembly, alternative splicing

DATA DESCRIPTION

Background

Unique amongst the mammalian species as they are the only order with true powered-flight capability, bats have served as a unique model for studying evolutionary adaptation and morphological innovations, such as flight, echolocation and longevity [1, 2]. More recently, bats have been increasingly recognized as an important reservoir harboring numerous pathogenic viruses while displaying minimal clinical signs of disease [3]. Indeed, comparing the genomes of bats with other mammalian species has revealed an unexpected concentration of positively selected genes. These include those involved in DNA damage repair and innate immune functions, which may partially explain bats' unique tolerance to deadly viruses and their unusually long lifespan [4]. Together, this highlights bats as an emerging model organism in the study of ecology, development, aging and evolution.

Accurate assembly and annotation of genomes is a critical first step for further functional studies of genetic variation. To date, there are 14 draft bat genomes that are

published and deposited in NCBI (*Eidolon helvum*, *Eptesicus fuscus*, *Hipposideros armiger*, *Megaderma lyra*, *Miniopterus natalensis*, *Myotis brandtii*, *Myotis davidii*, *Myotis lucifugus*, *Pteropus alecto*, *Pteronotus parnellii*, *Pteropus vampyrus*, *Rhinolophus ferrumequinum*, *Rhinolophus sinicus*, *Rousettus aegyptiacus*, Table S1) [2, 4-7]. Most of these genomes were assembled using only short illumina® sequencing reads (49-150 bp), with the exception of *R. aegyptiacus*, which utilized both short reads (data not released) from the illumina® HiSeq platform and long reads (data not released) from the PacBio® platform, resulting in a hybrid genome assembly. The long-read length of PacBio® sequencing, which is available for both DNA and RNA sequencing (also known as Iso-Seq), has shown considerable promise in genomics studies. For example, PacBio® DNA sequencing has improved assembly of the human [8], gorilla [9], loblolly pine [10] and avian genomes [11], while Iso-Seq has helped deepen our understanding of alternative splicing in the chicken [12], coffee bean [13] and maize [14] transcriptomes.

To produce a reliable genome resource and more thoroughly annotated genome than that of other bats, we employed the PacBio® technology to sequence both the genome and transcriptome of the cave nectar bat (also known as common nectar bat, dawn bat, common dawn bat, and lesser dawn bat), *Eonycteris spelaea* (*E. spelaea*, NCBI Taxonomy ID: 58065). We hoped the PacBio long-read technology would facilitate accuracy in annotating this evolutionary divergent species. *E. spelaea* is a specialist nectar-feeding bat that feeds predominantly on nectar and pollen, and is widely distributed over both the tropics and subtropics, throughout the Indomalayan region [15-17]. This species has been associated with pollination of durians and other fruits of both

cultural and economic importance throughout Asia [18]. Additionally, this species has been identified as a carrier of Orthoreoviruses, Lyssa Virus, Filoviruses, Flavivirus, Coronaviruses and Astroviruses [19-26]. The spread and abundance of this species make it an ideal subject for research purposes.

This newly assembled bat genome is 1.97 Gb in length, consisting of 4,470 sequences with a contig N50 of 8.0 Mbp. Identified repeat elements (REs) covered 34.65% of the genome and a total of 20,640 protein coding genes were annotated. Also, 29,493 alternative spliceosomes for 10,607 genes were identified. Together, this resource and the identified regulatory elements provide information on the functional roles and relationships of various genomic loci, which in turn can be comparatively analyzed to further understand a vast array of bat-specific physiological features.

Sampling and Sequencing

For whole genome sequencing, a total of 175 SMRT cells were sequenced. This yielded 15,518,413 (~161 Gb) reads with a mean sub-read length of 10,381 bp (standard deviation, SD, 7,424) and a N50 read length of 14,941 bp (Table 1). This translated into an ~80x coverage for the target genome (~2 Gb in length: estimates based on the average size of all previously sequenced 14 bat genomes, Figure S1). The ~80x coverage is above the minimum 50-60x coverage reported for self-correction of the high-error PacBio reads compared to Illumina reads [27].

For isoform sequencing (Iso-Seq), 357,722 (~1 Gb in total length) raw sub-reads (read length mean ± SD, 2,934 ± 1,775) were obtained (Table 1), representing an

~20x coverage of the bat transcriptome repertoire (estimated using *P. alecto*'s 21,593 unique annotated genes, accounting for ~0.05 Gb in length). After length-filtering and duplicate-collapsing (see Methods), 31,639 unique full-length (FL) transcripts were used for further subsequent analysis and gene annotation.

Genome Assembly and Evaluation

After several rounds of parameters adjustment with the Falcon (v. 0.3.0) algorithm (see Methods), we obtained a final 1.97 Gb assembly (named Espe.v1) which consists of 4,470 sequences with a contig N50 of 8.0 Mb (Table 2). We employed the BUSCO (v. 3) method [28] to evaluate the completeness of the genome annotation. The result showed that the vast majority (92.8%) of the representative mammal gene set (mammalia_odb9, which contains 4,104 single-copy genes that are highly conserved in mammals) was present in the assembled bat genome, demonstrating the completeness of gene set identification (Table 2). The GC content was 40.3%, similar to those of *P. alecto* (39.7%) and *R. aegyptiacus* (40.2%, Table 2). Overall, these metrics compare well with other recently published bat genomes, confirming Espe.v1 to be a reliable substrate for further genomic analyses.

Genome Annotation

RepeatMasker (v. 4.0.6) [29] was conducted with RMBlast (v. 2.2.28) to mask all the known mammalian transposon-derived REs. In order to compare the REs of *E. spelaea* with that of other genomes, we also performed the same analysis on all 14 available bat genomes on NCBI. Consistent with observations that PacBio® technology is a better

solution for solving repeats [8, 27], we found that known REs accounted for 34.65% of the genome in Espe.v1, which is the highest proportion amongst all bat genomes published to date (0.71% higher than that of the second RE abundant bat genome, *R. sinicus*, Table 2, Figure S1). Similar to other bat genomes, the long interspersed nuclear elements (LINEs) and long terminal repeat (LTR) elements constituted two of the highest proportion of all REs in Espe.v1, 51.32% and 18.05%, respectively (Table S2).

After repeat masking, the genome was annotated with Maker2 (v. 2.31.9) [30] by integrating homologous prediction, *ab initio* prediction and Iso-Seq-based prediction methods (see Methods). As a result, the predicted gene set included 20,640 protein-coding genes (Figure 1), of which 11,819 (57.2%) unique coding genes were supported by Iso-Seq and 16,637 (80.6%) were supported by homologous predictions. The relatively higher number of protein coding genes predicted in *E. spelaea* compared to *P. alecto* and *R. aegyptiacus* is likely due to a more homologous approach being used to predict the gene models of *E. spelaea* than that of *P. alecto* and *R. aegyptiacus* (see Method). In summary, 18,588 (90.1%) of the coding genes were supported by at least two types of prediction evidence (homologous and Iso-Seq evidence).

Phylogenetic Analysis

To evaluate the similarities and differences of available bat genomes and their evolutionary relationship, it is necessary to compare the phylogenies of *E. spelaea* to other bats and other mammalian species**.** Despite significant biological differences in the behaviours of bats, genetically some species are phylogenetically close and this may impact any study of the homology between species. We identified single-copy

orthologous gene clusters from 13 published genomes (eight bat genomes: *M. brandtii*, *M. lucifugus*, *M. davidii*, *E. spelaea*, *E. fuscus*, *P. alecto*, *P. vampyrus*, and *R. aegyptiacus*, five other mammalian genomes: *Homo sapiens*, *Mus musculus*, *Bos taurus*, and *Equus caballus* and *Monodelphis domestica*; with *M. domestica* as the outgroup. Table S1) using the Proteinortho software [31]. In total, 3,185 single-copy gene families across all 13 species were identified.

The divergence times of *E. spelaea* and the 11 mammals (excluding *M. domestica*) were estimated using 999,609 four-fold degenerate sites from the 3,185 single-copy genes. The topological order and estimated divergence time of our phylogeny analysis (Figure 2) is consistent with previous studies, with bats, *E*. *caballus* (horse) and *B*. *taurus* (cow) clustering together within the Laurasiatheria superorder (bats diverging ~79.95 million years ago [MYA]) [4]. Our analysis also revealed that *E. spelaea* was the closest sister taxon to *R. aegyptiacus*, an Egyptian fruit bat that is distributed throughout Africa [32]. The divergence time between these two bat species was estimated at ~20.36 MYA, indicating a relatively recent divergence. As *P. alecto*, *R. aegyptiacus* and *E. spelaea* are all relatively close on the phylogenetic tree, and all have reliable genome annotations, we focus on these three species for a more detailed comparison.

Iso-Seq analysis

One of the major advantages of the Iso-Seq technology is that it captures full length (FL) gene isoforms without the need for any downstream assembly. The large number of unique transcripts recovered though Iso-Seq enabled us to make a general assessment

of transcriptional complexity of the bat genome. Of the 31,639 FL Iso-Seq transcripts, 382 RE transcripts (98 LTRs, 31 DNA elements, 7 satellites, 244 LINEs, 1 short interspersed nuclear elements (SINEs), and 1 unknown RE) were filtered out from further analysis using RepeatMasker (see Methods). The remaining 31,257 clean transcripts were compared against our homology and *ab initio* predicted genes. Out of the 20,640 coding genes, we observed 10,033 (5,925 are supported by clean Iso-Seq transcripts) single transcript genes and 10,607 (5,894 are supported by clean Iso-Seq transcripts) alternatively spliced genes. Overall, we found an isoform to gene ratio of 1.92 (39,526 transcripts per 20,640 genes) in *E. spelaea*, which is lower than 3.62 (167,430 transcripts per 46,298 genes) in human, but higher than 1.49 (33,093 transcripts per 22,264 genes) in *P. alecto* (Figure 3A). When narrowed down to genes only observed by Iso-Seq, we found an isoform to gene ratio of 2.39 (28,289 transcripts per 11,819 genes), suggesting that the PacBio® Iso-Seq technology has significantly increased the isoform diversity discovery of *E. spelaea*'s transcriptome compared to that of *P. alecto*'s transcriptome, which was sequenced using the illumina® RNASeq technology (Fisher Exact Test, p-value < 10^{-5}).

The alternative transcript events were further classified into Skipping Exon (SE), Alternative 5'/3' Splice Sites (A5/A3), Mutually Exclusive Exons (MX), Retained Intron (RI) and Alternative First/Last Exons (AF/AL) by SUPPA software (Last updated 02/07/2017) [33]. We identified 30,487 alternative splicing events in the Iso-Seq dataset, which is 5.80-fold lower than that in human but 1.62-fold higher than that in *P. alecto* (Figure 3B). In particular, alternative 5'- (7,783 events) and 3'- (11,258 events) splicing were two of the most predominant events in the *E. spelaea* spliceosome repertoire

(Figure 3B). Our results provide the first comprehensive overview of splice variants in any bat species using a direct sequencing analysis approach rather than *in silico* analysis.

Conclusion and Discussion

In this study, we provided the first assembly of a bat genome solely using the PacBio® long-read sequencing technology. The *E. spelaea* genome assembly exemplifies the power of long-read sequencing technologies in rapid *de novo* assembly of a non-model genome and alternative-splicing isoform identification. Compared to the hybrid assembled *R. aegyptiacus* genome, the "PacBio-only" assembly was faster, more straightforward and possessed higher contiguous N50 contigs and better repeat-resolving with limited gene integrity loss (combined, there are 0.7% more fragmented or missing genes than that of *R. aegyptiacus* in the BUSCO report, Table 2). This slight decrease in gene integrity is probably due to the high depth of short read sequencing, which provided a more complete and uniform coverage across all genome, overall resulting in a better coverage on the gene content. Thus, our study provides a high-quality reference genome for use in any future comparative studies. Even without scaffolding, these highly contiguous contigs and FL gene transcripts will be helpful to researchers to extract more accurate genomic loci information of their genes of interest, saving a great amount of energy, resources and time. Our Iso-Seq results have increased our understanding of the complexity of the bat transcriptome and aided in alternative transcript identification. This complexity in the transcriptome of bats is still likely to be underestimated since Iso-Seq was performed at a relatively shallow depth

(~20x coverage) in this study, therefore lowly expressed transcripts could have been missed due to the lack of depth. We would like to further highlight that this complexity is attributed by the type and number of alternative transcription events, as well as previously unannotated transcripts in bats. As more and more transcriptome data becomes available for *E. spelaea*, e.g. Illumina transcriptome sequencing of various tissues, this will aid in the identification of other alternative transcripts and continuously improve the annotation of the genome and the accuracy of protein coding sequences, as has been evident with the *P. alecto* genome since its first release. Taken together, we have provided a valuable resource, an *E. spelaea* genome and transcriptome database, for future comparative and functional studies, as well as demonstrated the advantages of employing the latest long-read sequencing technology in such studies of exotic species.

METHODS

Bat Sample Processing

Eonycteris spelaea was captured in Singapore at dusk using mist nets and transferred to clean customized bat bags for transportation. All animal processing work was conducted in accordance with approved guidelines and methods in line with permits obtained from the National Parks Board, Singapore (NP/RP14-109) and animal ethics approval from the National University of Singapore (B16-0159). Bats were euthanized using isoflurane and exsanguinated via cardiac bleed. Various tissue samples, as detailed in Table S3, were harvested and preserved in RNA*later*™ Stabilization Solution (Invitrogen™). Tissues were homogenized and RNA was extracted using RNeasy® Mini

Kit (Qiagen) with an additional on-column DNase digestion step using RNase-Free DNase Set (Qiagen). Extracted RNA was subsequently eluted in RNase-Free water and stored at -80 ℃.

For genomic DNA extraction, fresh lung and kidney samples were snap frozen in liquid nitrogen immediately upon harvesting and pounded into powder form before extraction using the Gentra Puregene Tissue Kit (Qiagen).

DNA and Iso-Seq sequencing

Genomic DNA was extracted from a single male *E. spelaea*. Two DNA libraries, derived from kidney and lung samples, were constructed using SMRTbell Template Prep Kits 1.0 (Pacific Biosciences) with a 20 kb insert size. Single-molecule, real-time (SMRT) sequence data were generated using P6v2 polymerase binding and C4 chemistry (P6-C4) kits over a 6-hour movie run-time on the PacBio® RSII instrument. Library construction and sequencing runs were performed by a commercial sequencing provider DNA Link Inc. (Korea).

For Iso-Seq sequencing, total RNA from a panel of tissues (Table S3) was extracted from two individuals, a female and a male. Tissue RNA from individual bats was then pooled together. Four libraries of 1–2 kb, 2–3 kb, 3–6 kb and 5-10 kb insert sizes were generated using the P6-C4 kits and subsequently sequenced. In total, 34 SMRT cells were sequenced on the PacBio RS II platform over a 3 to 4-hour movie run time. Library construction and Iso-Seq runs were performed at the Duke-NUS Genome Biology Facility (Duke-NUS Medical School, Singapore).

Genome Assembly

PacBio® sub-reads were filtered with default parameters and submitted to Falcon (https://github.com/PacificBiosciences/FALCON, v. 0.3.0) for genome assembly. For the final assembly (Espe.v1), a total of 15,518,413 sub-reads (read length mean ± SD, 10,382 ± 7,424) were used for assembly, with a length-cutoff parameter of 2 kb for initial mapping to build pre-assembled reads (also referred as error-corrected preads). The pre-assembly module is a built-in module in Falcon for error-correcting PacBio sub-reads. A total of 15,470,844 preads were generated (read length mean ± SD, 7,034 ± 5,874) from the PacBio sub-reads. Preads over 10 kb were used (length-cutoff-pr) to seed pre-assembly. Daligner was used to detect all pairwise local overlapping region between sub-reads and also between corrected preads. Overlapping options were set to "-v -B128 -M40 -e.70 -l2000 -s400" for pre-assembly and "-v -B128 -M40 -h45 -e.96 -l500 -s400" for alignment of corrected preads. To reduce computation and assembly graph complexity, overlaps build by corrected preads were filtered by "--max-diff 300 --max-cov 400 --min-cov 2 --bestn 20" to remove the transitive reducible overlaps. Consensus were built from the overlapping preads on "--output-multi --min-idt 0.70 --min-cov --max-n-read 400". Finally, Primary and associated contigs were polished using Quiver with default parameters.

Iso-Seq Analysis

For Iso-Seq analysis, raw reads were classified into Circular Consensus Sequences (CCS) and non-CCS sub-reads by ToFu (v. 4.1) [34], and FL CCS reads were filtered out if both the 5'- and 3'-cDNA primers were present, as well as a polyA tail signal

preceding the 3'-primer. To improve consensus accuracy, the isoform-level clustering algorithm ICE (Iterative Clustering for Error Correction) and Quiver were applied to generated FL transcripts with >= 99% post-correction accuracy. Next, the Quiver-polished FL CCS reads were mapped to the assembled genome using GMAP (v. 2018-01-26) [35] and collapsed by the pbtranscript-ToFU package (http://github.com/PacificBiosciences/cDNA-primer/, last updated: 10/15/2015) with default parameters to collapse redundant transcripts. Collapsed transcripts were screened for REs by RepeatMasker (v. open-4.0.6) [36] to mask all mammalian RE sequences. Transcripts with >= 70% bases masked were denoted as REs and discarded from further analysis. Alternative splicing events in the repeat-cleaned Iso-Seq reads, human (Ensembl GRCh38.p10) and *P. alecto* (NCBI assembly ASM32557v1) mRNAs were classified with SUPPA (Last updated 02/07/2017) under default parameters.

Genome Annotation

Maker2 (v. 2.31.9) [30] was utilized to perform genome annotation. Repetitive genomic elements were identified and masked from annotation with RepeatMasker using the Repbase database (Update 20160829) [37]. Cleaned Iso-Seq transcripts (see above) were used as transcript evidence. Augustus (v. 2.7) [38] and SNAP (Release 11/29/2013) [39] were used as *ab initio* gene predictors. Unique protein sequences from eight different mammals (*B. taurus*, *Canis familiaris*, *E. caballus*, *H. sapiens*, *M. musculus*, *M. lucifugus*, *P. alecto*, *P. vampyrus*, Table S1) were downloaded from Ensembl (last accessed: 5/15/2017) [40] and used for homology-based prediction. The

Maker2 pipeline was first run on the masked genome using the Iso-Seq transcriptome to infer gene predictions (est2genome = 1), and training files for the *ab initio* gene predictors Augustus and SNAP were generated based on these results. Then, the annotation pipeline was run iteratively two additional times using the Iso-Seq transcriptome as evidence (est2genome = 0) and providing new training files with each run. At this point, the protein-homology evidence was set to include all unique proteins in the eight different mammals. Next, Maker predict transcripts were merged with Iso-Seq transcripts and collapsed using pbtranscript-ToFU to include all the unique alternative spliced transcripts. Finally, low-quality genes shorter than 50 amino acids and/or exhibiting premature termination were removed to produce the final gene set.

Phylogenetic Analysis

Phylogenetic tree construction and divergence time estimation were performed as described [4]. Briefly, Proteinortho software (v. 5.16b) [31] was used to identify the single-copy orthologous genes under default parameter setting. Using *M. domestica* as an outgroup, we identified 3,185 single-copy orthologous gene from *E. spelaea* and 11 other mammalian genomes (as described above). Coding sequence (CDS) from each single-copy family was aligned by MUSCLE (v. 3.8.31) [41]. Four-fold degenerate sites were extracted from each gene alignments by an in-house Python script and concatenated to one super gene for each species. Then, RAxML (v. 8.2.11) [42] was applied to build phylogenetic trees for the concatenated sequences as described [4]. 1,000 bootstrap replicates were employed to assess branch reliability in RAxML. Lastly, PAML (v. 4.9c) mcmctree [43] was used to determine split times based on the topology

obtained in the RAxML analysis [4]. The gamma prior for the overall substitution rate was described by shape and scale parameters which were set as 1 and 11.1 respectively, calculated according to the substitution rate per time unit using PAML baseml [44]. Fossil calibrations were retrieved from the TimeTree database (last accessed: 12/15/2017) [45]. Other parameters were set as default. PAML mcmctree pipeline was run two independent times to confirm convergence and all acceptance proportions fall in the interval (20%, 40%).

Availability of Supporting Data

Genome data is available in project accession PRJNA427241 in the NCBI database. Further supporting data can be found in the GigaScience GigaDB [46].

Competing Interests

The authors declare that they have no competing interests.

Author Contributions

L-F.W. and J.H.J.N. conceived and designed the study; I.H.M, B.P.Y-H.L., C.Y.T and J.H.J.N. led the bat field work; C.Y.T and J.H.J.N. performed the experimental processing of samples; M.W. led the sequence analysis; F.Z. and A.T.I. contributed to data analysis. All authors contributed to manuscript writing, read and approved the final version for submission.

Acknowledgments

This work was funded by the Singapore National Research Foundation Competitive Research Programme grant (NRF2012NRF-CRP001-056). AT Irving is supported by a New Investigator's Grant from the National Medical Research Council of Singapore (NMRC/BNIG/2040/2015). IH Mendenhall was supported by a New Investigator's Grant from the National Medical Research Council of Singapore (NMRC/BNIG/2005/2013). BPYH Lee was supported by a research grant from the Wildlife Reserves Singapore Conservation Fund (WRSCF). We thank Ms Dolyce Low Hong Wen, Ms Erica Sena Neves and Ms Sophie Alison Borthwick for their help with bat field work, the Duke-NUS Genome Biology Facility and the Genome Institute of Singapore for library construction, quality control, sequencing and data delivery, and the Duke-NUS High Performance Computing infrastructure for computational resources.

Reference:

1. Simmons, N.B., et al., *Primitive Early Eocene bat from Wyoming and the evolution of flight and echolocation.* Nature, 2008. **451**(7180): p. 818-21.
2. Seim, I., et al., *Genome analysis reveals insights into physiology and longevity of the Brandt's bat Myotis brandtii.* Nat Commun, 2013. **4**: p. 2212.
3. Olival, K.J., et al., *Host and viral traits predict zoonotic spillover from mammals.* Nature, 2017. **546**(7660): p. 646-650.
4. Zhang, G., et al., *Comparative analysis of bat genomes provides insight into the evolution of flight and immunity.* Science, 2013. **339**(6118): p. 456-60.
5. Eckalbar, W.L., et al., *Transcriptomic and epigenomic characterization of the developing bat wing.* Nat Genet, 2016. **48**(5): p. 528-36.
6. Dong, D., et al., *The Genomes of Two Bat Species with Long Constant Frequency Echolocation Calls.* Mol Biol Evol, 2017. **34**(1): p. 20-34.
7. Parker, J., et al., *Genome-wide signatures of convergent evolution in echolocating mammals.* Nature, 2013. **502**(7470): p. 228-31.

8. Pendleton, M., et al., *Assembly and diploid architecture of an individual human genome via single-molecule technologies.* Nat Methods, 2015. **12**(8): p. 780-6.
9. Gordon, D., et al., *Long-read sequence assembly of the gorilla genome.* Science, 2016. **352**(6281): p. aae0344.
10. Zimin, A.V., et al., *An improved assembly of the loblolly pine mega-genome using long-read single-molecule sequencing.* Gigascience, 2017. **6**(1): p. 1-4.
11. Korlach, J., et al., *De novo PacBio long-read and phased avian genome assemblies correct and add to reference genes generated with intermediate and short reads.* Gigascience, 2017. **6**(10): p. 1-16.
12. Kuo, R.I., et al., *Normalized long read RNA sequencing in chicken reveals transcriptome complexity similar to human.* BMC Genomics, 2017. **18**(1): p. 323.
13. Cheng, B., A. Furtado, and R.J. Henry, *Long-read sequencing of the coffee bean transcriptome reveals the diversity of full-length transcripts.* Gigascience, 2017. **6**(11): p. 1-13.
14. Wang, B., et al., *Unveiling the complexity of the maize transcriptome by single-molecule long-read sequencing.* Nat Commun, 2016. **7**: p. 11708.
15. Ghanem, S.J. and C.C. Voigt, *Increasing Awareness of Ecosystem Services Provided by Bats.* Advances in the Study of Behavior, Vol 44, 2012. **44**: p. 279-302.
16. Shao, W.W., et al., *Characterization of microsatellite loci in the lesser dawn bat (Eonycteris spelaea).* Mol Ecol Resour, 2008. **8**(3): p. 695-7.
17. Francis, C.M. and P. Barrett, *A guide to the mammals of Southeast Asia*. 2008, Princeton, N.J.: Princeton University Press.
18. Bumrungsri, S., et al., *The pollination ecology of durian (Durio zibethinus, Bombacaceae) in southern Thailand.* Journal of Tropical Ecology, 2009. **25**(1): p. 85-92.
19. Laing, E.D., et al., *Serologic Evidence of Fruit Bat Exposure to Filoviruses, Singapore, 2011-2016.* Emerg Infect Dis, 2018. **24**(1): p. 114-117.
20. Mendenhall, I.H., et al., *Identification of a Lineage D Betacoronavirus in Cave Nectar Bats (Eonycteris spelaea) in Singapore and an Overview of Lineage D Reservoir Ecology in SE Asian Bats.* Transbound Emerg Dis, 2017. **64**(6): p. 1790-1800.
21. Mendenhall, I.H., et al., *Influence of age and body condition on astrovirus infection of bats in Singapore: An evolutionary and epidemiological analysis.* One Health, 2017. **4**: p. 27-33.
22. Yang, X.L., et al., *Genetically Diverse Filoviruses in Rousettus and Eonycteris spp. Bats, China, 2009 and 2015.* Emerg Infect Dis, 2017. **23**(3): p. 482-486.

23. Kapoor, A., et al., *Use of nucleotide composition analysis to infer hosts for three novel picorna-like viruses.* J Virol, 2010. **84**(19): p. 10322-8
24. Lumlertdacha, B., et al., *Survey for bat lyssaviruses, Thailand.* Emerg Infect Dis, 2005. **11**(2): p. 232-6.
25. Taniguchi, S., et al., *First isolation and characterization of pteropine orthoreoviruses in fruit bats in the Philippines.* Arch Virol, 2017. **162**(6): p. 1529-1539.
26. Varelas-Wesley, I. and C.H. Calisher, *Antigenic relationships of flaviviruses with undetermined arthropod-borne status.* Am J Trop Med Hyg, 1982. **31**(6): p. 1273-84.
27. Berlin, K., et al., *Assembling large genomes with single-molecule sequencing and locality-sensitive hashing.* Nat Biotechnol, 2015. **33**(6): p. 623-30.
28. Simao, F.A., et al., *BUSCO: assessing genome assembly and annotation completeness with single-copy orthologs.* Bioinformatics, 2015. **31**(19): p. 3210-2.
29. Tarailo-Graovac, M. and N. Chen, *Using RepeatMasker to identify repetitive elements in genomic sequences.* Curr Protoc Bioinformatics, 2009. **Chapter 4**: p. Unit 4 10.
30. Holt, C. and M. Yandell, *MAKER2: an annotation pipeline and genome-database management tool for second-generation genome projects.* BMC Bioinformatics, 2011. **12**: p. 491.
31. Lechner, M., et al., *Proteinortho: detection of (co-)orthologs in large-scale analysis.* BMC Bioinformatics, 2011. **12**: p. 124.
32. Lučan, R.K., et al., *Reproductive seasonality of the Egyptian fruit bat (Rousettus aegyptiacus) at the northern limits of its distribution.* Journal of Mammalogy, 2014. **95**(5): p. 1036-1042.
33. Alamancos, G.P., et al., *Leveraging transcript quantification for fast computation of alternative splicing profiles.* RNA, 2015. **21**(9): p. 1521-31.
34. Gordon, S.P., et al., *Widespread Polycistronic Transcripts in Fungi Revealed by Single-Molecule mRNA Sequencing.* PLoS One, 2015. **10**(7): p. e0132628.
35. Wu, T.D. and C.K. Watanabe, *GMAP: a genomic mapping and alignment program for mRNA and EST sequences.* Bioinformatics, 2005. **21**(9): p. 1859-75.
36. Smit, A., R. Hubley, and P. Green, *RepeatMasker Open-4.0. 2015.* Google Scholar, 2016.
37. Bao, W., K.K. Kojima, and O. Kohany, *Repbase Update, a database of repetitive elements in eukaryotic genomes.* Mob DNA, 2015. **6**: p. 11.

38. Stanke, M., et al., *AUGUSTUS: a web server for gene finding in eukaryotes.* Nucleic Acids Res, 2004. **32**(Web Server issue): p. W309-12.
39. Korf, I., *Gene finding in novel genomes.* BMC Bioinformatics, 2004. **5**: p. 59.
40. Zerbino, D.R., et al., *Ensembl 2018.* Nucleic Acids Res, 2017.
41. Edgar, R.C., *MUSCLE: multiple sequence alignment with high accuracy and high throughput.* Nucleic Acids Res, 2004. **32**(5): p. 1792-7.
42. Stamatakis, A., *RAxML version 8: a tool for phylogenetic analysis and post-analysis of large phylogenies.* Bioinformatics, 2014. **30**(9): p. 1312-3.
43. dos Reis, M. and Z. Yang, *Approximate likelihood calculation on a phylogeny for Bayesian estimation of divergence times.* Mol Biol Evol, 2011. **28**(7): p. 2161-72.
44. Yang, Z., *PAML 4: phylogenetic analysis by maximum likelihood.* Mol Biol Evol, 2007. **24**(8): p. 1586-91.
45. Hedges, S.B., J. Dudley, and S. Kumar, *TimeTree: a public knowledge-base of divergence times among organisms.* Bioinformatics, 2006. **22**(23): p. 2971-2.
46. Sneddon, T.P., P. Li, and S.C. Edmunds, *GigaDB: announcing the GigaScience database.* Gigascience, 2012. **1**(1): p. 11.

Figure Legends

Figure 1. Venn diagram for coding gene predictions based on evidence sources. The different colours indicate various sources of evidence, and the values reflect the number of genes supported by each type of evidence.

Figure 2. Maximum-likelihood phylogenetic analysis of 3,185 genes in bats and mammalian species. The estimated divergence time (100 million years ago; MYA) is given at the nodes, with the 95% confidence intervals in parentheses. *M. domestica*, used as an outgroup species, was excluded in this figure.

Figure 3. The alternative splicing of *E. spelaea*'s coding genes. **A**. Comparison of number of alternative transcripts per annotated gene between *H. sapiens*, *P. alecto*, *E. spelaea* and PacBio® Iso-Seq *E. spelaea* transcriptomes. **B.** Comparison of rate of occurrence for the different classes of alternative transcripts between *H. sapiens*, *P. alecto*, and the *E. spelaea* PacBio® Iso-Seq transcriptome. Abbreviations: A5/A3, Alternative 5'/3' Splice Sites; AF/AL, Alternative First/Last Exons; MX, Mutually Exclusive Exons; RI, Retained Intron; SE, Skipping Exon.

Table 1: Data counts and library information for the *E. spelaean* genome

Library type	Insert size	No. of subreads	N50 size	Total bp
DNA sequencing	20 kb	15,518,413	14,941	161,109,271,053
Iso-Seq	1–2 kb	107,230	1,409	148,990,235
	2–3 kb	95,170	2,299	226,856,499
	3–6 kb	104,687	3,700	403,584,865
	5–10 kb	50,635	5,673	270,216,738
	Total	357,722	3,557	1,049,648,337

Table 2. Comparison of genome features between *E. spelaea*, *P. alecto* and *R. aegyptiacus*.

Type	*E. spelaea*	*P. alecto*	*R. aegyptiacus*
Sequencing technology	PacBio	Illumina HiSeq	Illumina HiSeq + PacBio
Genome coverage	~83x	~110x	~169x
Total Genome Length (bp)	1,966,861,576	1,985,975,446	1,910,250,568
Number of Contig/Scaffold	4,470/N.A.	170,164/65,598	3,049/2,490
Contig/Scaffold N50 (bp)	8,002,591/N.A.	31,841/15,954,802	1,488,988/2,007,187
GC level	40.3 %	39.7 %	40.02 %
Repetitive Elements[1]	34.65 %	30.08 %	29.58 %
Number of Coding Genes	20,640	18,363	19,668
BUSCO[2](n=4104)	C:92.8%,F:4.8%,M:2.4%	C:94.1%,F:3.7%,M:2.2%	C:93.5%,F:4.4%,M:2.1%

[1]Known mammalian repetitive elements deposit in Repbase (Update 20160829).
[2]BUSCO: Benchmarking Universal Single-Copy Orthologs; C: Complete BUSCOs; F: Fragmented BUSCOs; M: Missing BUSCOs; N.A. not available.

Figure 1

Click here to access/download;Figure;Figure 1 180725.docx

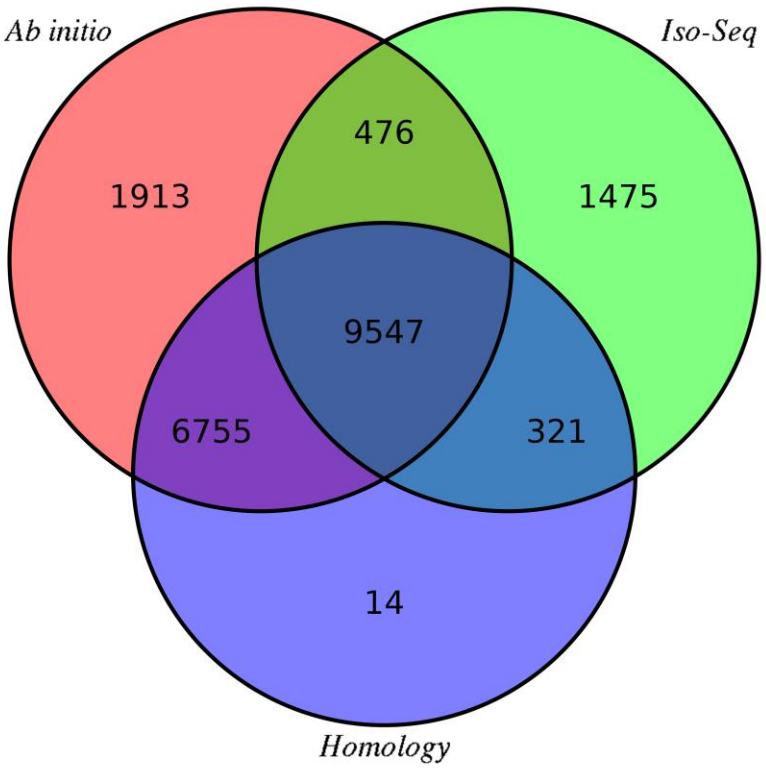

Click here to access/download;Figure;Figure 2 180725.docx

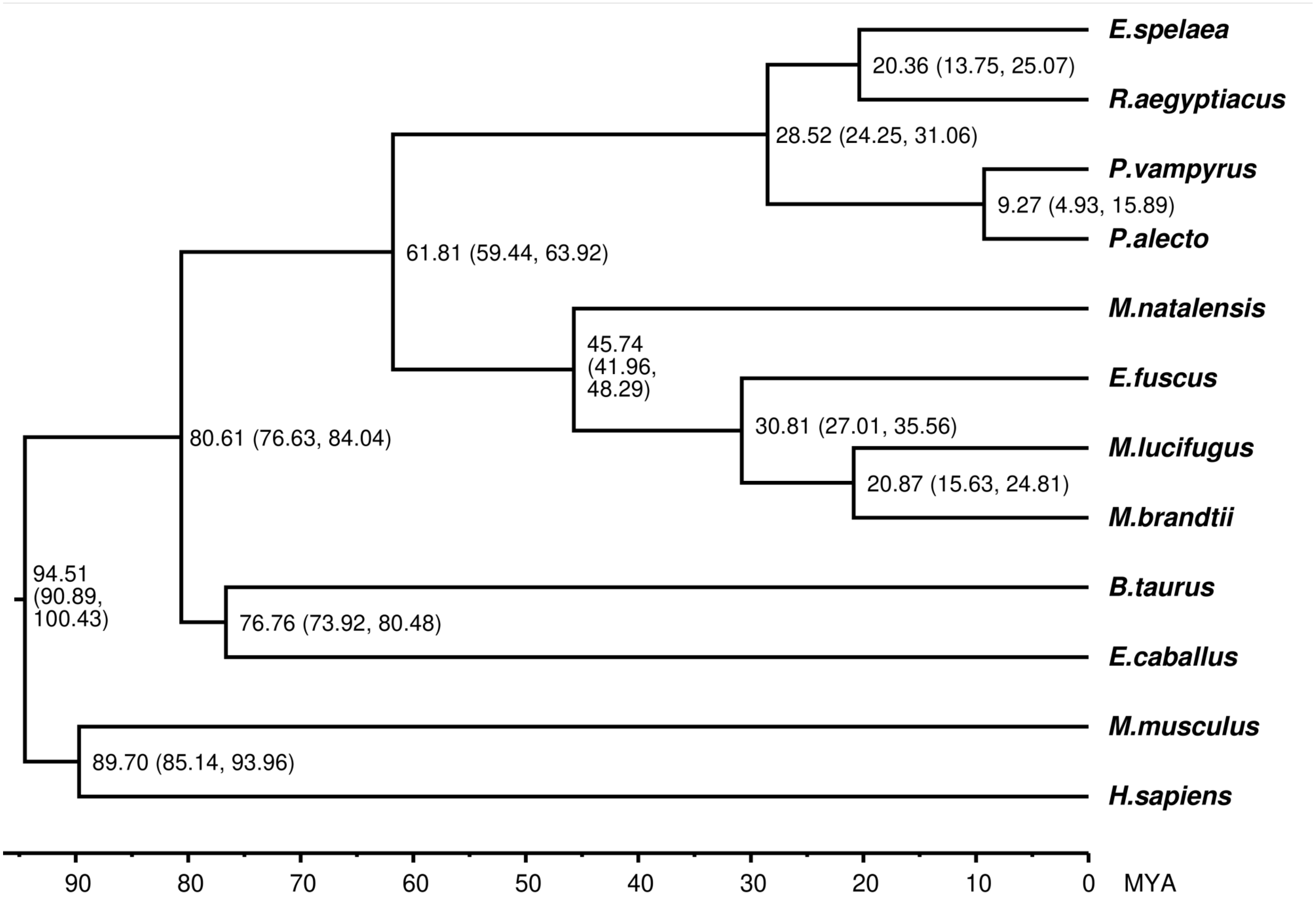

Figure 3

Click here to access/download;Figure;Figure 3 180725.docx

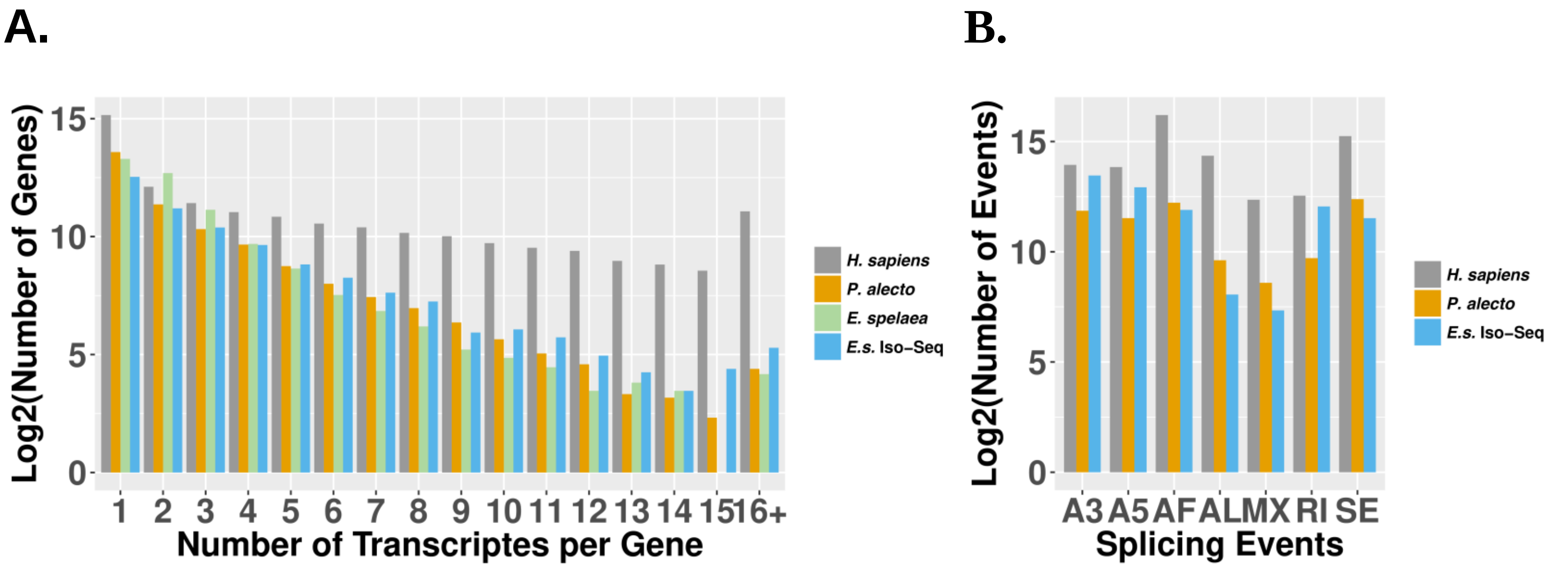

Click here to access/download
Supplementary Material
GigaSci-Es genome-SI-Submission-180725.docx
