# Supplementary material for: Exploring the genome and transcriptome of the cave nectar bat Eonycteris spelaea with PacBio long-read sequencing
Source: Gigascience. 2018 Sep 20;7(10):giy116. doi: 10.1093/gigascience/giy116 (PMC6177735; doi:10.1093/gigascience/giy116)
Supplement: Reviewer_3_Original_Submission_(Attachment).pdf [file giy116_reviewer_3_original_submission_(attachment).pdf]

Comments to the Authors

The authors have sequenced and assembled the genome and transcriptome of the bat *E. spelaea*, and analyzed its composition in comparison to other bat species. This will be a valuable resource for future comparative genomics studies, and a useful blueprint for future genome projects. However, I have one or two major points that I feel are worthy of further exploration before publication.

Reviewer's comments

Major points

1) P.5 lines 101 – 107, 112 -119: Given the intense details of these sections, they read more like a specific set of materials and methods, and should be placed in the appropriate section

2) I am a little concerned about the usage of *D. mealogaster* as an out-group for the author's phylogenetic analyses. Their datasets used to analyze phylogeny contains 12 mammalian genomes and 1 arthropod. I am worried that this may affect divergence time estimates. Perhaps the authors should consider using vertebrate taxon as an out-group, or at least state their reasoning for using an invertebrate as an out-group for a mammalian phylogeny.

3) In Table 2, combining PacBio and Illumina reads results in less coding genes for *R. aegyptiacus* than either genome where only one method is utilized. This is despite the fact that there are very similar GC level and repetitive elements between *E. spelaea* and *R. aegyptiacus*, with both appearing as sister taxa in the described phylogeny. The authors should comment on, discuss, or explain such a stark difference (independent methods providing more coding genes than combined methods).

4) The authors have missed out on an opportunity here to comment on the utility of PacBio long reads only in genome assembly (*E. spelaea*) compared to a genome assembly with both PacBio and Illumina reads (*R. aegyptiacus*), as this may have implications for researchers trying to find the optimum, most cost affective means of genome assembly. A short comment in the discussion should be included discussing this.

Minor points

p. 2, line 29: Authors use "aging", but use "ageing" in line 66. Please use consistent spelling.

p. 3, line 57: Perhaps the statement "Unique amongst the mammalian species and as the only group of mammals with true powered flight" would be better written as "Unique amongst mammalian species due to being the only order with true powered flight"

p. 3, line 62: Sentence used plural 'comparing' but past tense 'revealed', authors shoud say "has revealed".

p.3 line 62-65: very long, sentence. Consider breaking up.

p.4 Line 83. This sentences suggests only 1 bat genome exists, consider "annotate the genomes of other bats" as an alternative.

p. 6 line 128: should it be “we employed the BUSCO (v.3) method” rather than “employed BUSCO method”?

p. 6 line 132: change gene set**s** identification to gene set identification

p. 7 line 143: Known REs accounted for 34.65% of what? The genome? Or of all repetitive elements detected?

p. 7 line 144: Should Espe.v1 get a citation?

p. 8, line 169: should be “closest sister taxon to *R. aegyptiacus*” rather than “closest to”

p. 11, line 243: What length were the sub-reads?

p. 11, Genome assembly section: Could the authors provide a short single sentence description of what each software does, in addition to providing the parameters, to give greater context.
