# Supplementary material for: Exploring the genome and transcriptome of the cave nectar bat Eonycteris spelaea with PacBio long-read sequencing
Source: Gigascience. 2018 Sep 20;7(10):giy116. doi: 10.1093/gigascience/giy116 (PMC6177735; doi:10.1093/gigascience/giy116)
Supplement: Supplement File [file giy116_supplement_file.docx]

**Exploring the genome and transcriptome of the cave nectar bat *Eonycteris spelaea* with PacBio long-read sequencing**

*Ming Wen^1,†^, Justin H. J. Ng^1,†^, Feng Zhu, Yok Teng Chionh^1^, Wan Ni Chia^1^, Ian H. Mendenhall^1^, Benjamin P.Y-H. Lee^2^, Aaron T. Irving^1,*^,* *Lin-Fa Wang^1,*^*

**Supplementary Information**

**Figure S1.** Scatter plot of the genome size and proportion of repeat content for 15 bats genomes.


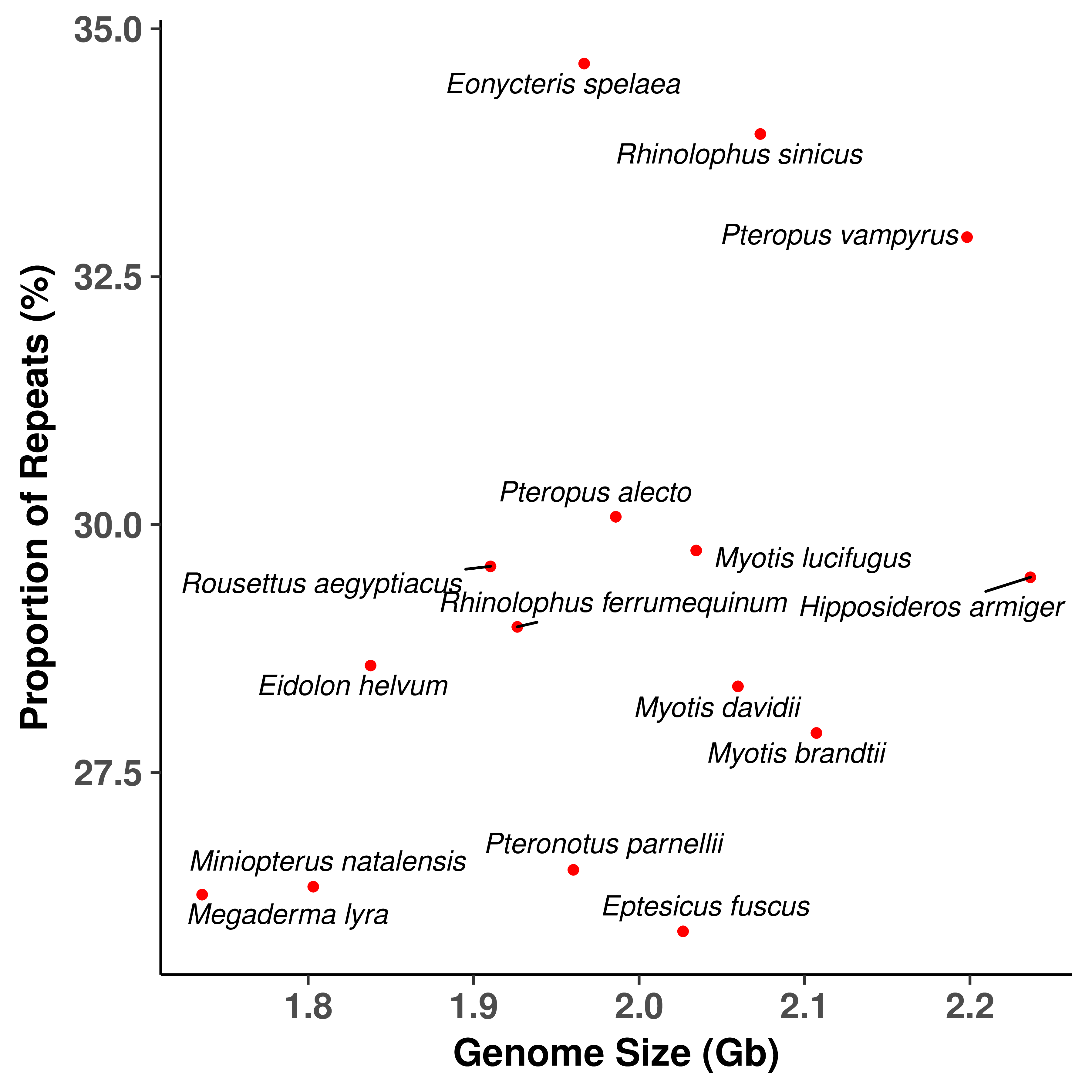


#

**Table S1:** Reference genomes used in this study.

| **Species** | **Genome build** | **Source** |
| --- | --- | --- |
| *Eidolon helvum* | ASM46528v1 | NCBI |
| *Eptesicus fuscus* | EptFus1.0 | NCBI |
| *Hipposideros armiger* | ASM189008v1 | NCBI |
| *Megaderma lyra* | ASM46534v1 | NCBI |
| *Miniopterus natalensis* | Mnat.v1 | NCBI |
| *Myotis brandtii* | ASM41265v1 | NCBI |
| *Myotis davidii* | ASM32734v1 | NCBI |
| *Myotis lucifugus* | Myoluc2.0 | NCBI |
| *Pteropus alecto* | ASM32557v1 | NCBI |
| *Pteronotus parnellii* | ASM46540v1 | NCBI |
| *Pteropus vampyrus* | Pvam_2.0 | NCBI |
| *Rhinolophus ferrumequinum* | ASM46549v1 | NCBI |
| *Rhinolophus sinicus* | ASM188883v1 | NCBI |
| *Rousettus aegyptiacus* | Raegyp2.0 | NCBI |
| *Bos taurus* | last accessed: 5/15/2017 | ENSEMBL |
| *Canis familiaris* | last accessed: 5/15/2017 | ENSEMBL |
| *Monodelphis domestica* | last accessed: 5/10/2018 | ENSEMBL |
| *Equus caballus* | last accessed: 5/15/2017 | ENSEMBL |
| *Homo sapiens* | last accessed: 5/15/2017 | ENSEMBL |
| *Mus musculus* | last accessed: 5/15/2017 | ENSEMBL |

**Table S2:** Descriptive statistics of the *E. spelaea* genome repeat elements using RepeatMasker. RepBase Update 20160829.

| **Repeat type** | **Number of elements*** | **Length occupied** | **Percentage of sequence** |
| --- | --- | --- | --- |
| SINEs: | 477797 | 66625129 bp | 3.39% |
| Alu/B1 | 9 | 512 bp | 0.00% |
| MIRs | 446217 | 62012063 bp | 3.15% |
| LINEs: | 770405 | 349687428 bp | 17.78% |
| LINE1 | 435579 | 263626673 bp | 13.40% |
| LINE2 | 286350 | 74757769 bp | 3.80% |
| L3/CR1 | 38139 | 8287380 bp | 0.42% |
| RTE | 9437 | 2875801 bp | 0.15% |
| LTR elements: | 354919 | 122966204 bp | 6.25% |
| ERVL | 80843 | 34754206 bp | 1.77% |
| ERVL-MaLRs | 131126 | 44982881 bp | 2.29% |
| ERV_classI | 75899 | 32054724 bp | 1.63% |
| ERV_classII | 45869 | 5780249 bp | 0.29% |
| DNA elements: | 358081 | 72837009 bp | 3.70% |
| hAT-Charlie | 199750 | 37609427 bp | 1.91% |
| TcMar-Tigger | 74602 | 18577533 bp | 0.94% |
| Unclassified: | 5114 | 961227 bp | 0.05% |
| Total interspersed repeats: |  | 613076997 bp | 31.17% |
| Small RNA: | 44 | 10639 bp | 0.00% |
| Satellites: | 76618 | 37652271 bp | 1.91% |
| Simple repeats: | 569564 | 25875094 bp | 1.32% |
| Low complexity: | 88087 | 4859257 bp | 0.25% |

* most repeats fragmented by insertions or deletions have been counted as one element

**Table S3:** Detailed listing of *E. spelaea* tissue RNA used for Iso-Seq analysis

| **Tissues** | **Amount of RNA (ng)** | |
| --- | --- | --- |
|  | **ES-002 (Male)** | **ES-04 (Female)** |
| Bladder | 300 | 300 |
| Brain | 300 | 300 |
| Eye | 100 | 100 |
| Heart | 100 | 100 |
| Intestine - Large | 300 | 300 |
| Intestine - Small | 300 | 300 |
| Kidney | 300 | 300 |
| Liver | 600 | 600 |
| Lung | 300 | 300 |
| Mammary Glands | - | 300 |
| Reproduction Tract | - | 300 |
| Salivary Gland | 300 | 300 |
| Spleen | 600 | 600 |
| Stomach | 300 | 300 |
| Testes | 300 | - |
| Thymus | 600 | 600 |
| Wing - Skin | 300 | 300 |
| Total Amount of RNA (ng) | 5,000 | 5,300 |
