# Supplementary material for: Exploring the genome and transcriptome of the cave nectar bat Eonycteris spelaea with PacBio long-read sequencing
Source: Gigascience. 2018 Sep 20;7(10):giy116. doi: 10.1093/gigascience/giy116 (PMC6177735; doi:10.1093/gigascience/giy116)
Supplement: giy116_Response_to_Reviewer_Comments_Report_(Original_Submission).pdf [file giy116_giy116_response_to_reviewer_comments_report_(original_submission).pdf]

Author's Response To Reviewer Comments

Close

List of responses attached in the cover letter

Close
