# Supplementary material for: Exploring the genome and transcriptome of the cave nectar bat Eonycteris spelaea with PacBio long-read sequencing
Source: Gigascience. 2018 Sep 20;7(10):giy116. doi: 10.1093/gigascience/giy116 (PMC6177735; doi:10.1093/gigascience/giy116)
Supplement: Response_to_Reviewer_Comments_Report_Revision_1.pdf [file giy116_response_to_reviewer_comments_report_revision_1.pdf]

Author's Response To Reviewer Comments

Close

Reviewer #2
Reviewer #2: Major comments:
When compared to the hybrid assembled R. aegyptiacus, the authors stated "combined, there are 0.7% more fragmented or missing genes than that of R. aegyptiacus in the BUSCO report". I cannot follow their explanation in the following sentence "This slight decrease in gene integrity……". Should be "the slight increase in gene integrity of R. aegyptiacus……"? please clarify this.

Reply: The original statement was correct, " there are 0.7% more fragmented or missing genes….", which resulted in "…slight decrease in gene integrity…". But it is presented in confusing way. Also, the 0.7% difference is very minimum and it might be better to not emphasize that in this context. We have modified the text to the following: "Compared to the hybrid assembled R. aegyptiacus genome, the "PacBio-only" assembly was faster, more straightforward and possessed higher contiguous N50 contigs and better repeat-resolving with a minimum difference (0.7%) in gene integrity".

Detailed comments:
Line 153, "18,588 (90.1%) of the coding genes were supported by at least two types of prediction evidences……" should be "18,588 (90.1%) of the coding genes were supported by at least one of the two types of prediction evidences……"?
Line 167, "……is consistent with previous studies" should be "……are consistent with previous studies". And please add references for previous studies.
Line 227, "therefore lowly expressed transcripts could have been missed due to the lack of depth" can be deleted here?
Line 231, "…… becomes ……" should be "… become ……"
Line 286, "…build…" should be "…built…"
Line 289, "… Primary…" should be "… primary…"
Line 334, "…orthologous gene…" should be "…orthologous genes…"
Line 337, "… gene alignments …" should be "… gene alignment …"

Reply: Thanks for the very detailed suggestions. All changes (reference addition) have been made accordingly.

Close
