# Supplementary material for: Exploring the genome and transcriptome of the cave nectar bat Eonycteris spelaea with PacBio long-read sequencing
Source: Gigascience. 2018 Sep 20;7(10):giy116. doi: 10.1093/gigascience/giy116 (PMC6177735; doi:10.1093/gigascience/giy116)
Supplement: Reviewer_1_Report_(Original_Submission) -- Michael Hiller — 4/20/2018 Reviewed [file giy116_reviewer_1_report_(original_submission).pdf]

Reviewer Report

Title: Exploring the genome and transcriptome of the cave nectar bat Eonycteris spelaea with PacBio long-read sequencing

Version: Original Submission **Date:** 4/20/2018

Reviewer name: Michael Hiller

Reviewer Comments to Author:

Wen et al. report a highly-contiguous contig assembly of the lesser dawn bat obtained with high-coverage PacBio long read data. They further used IsoSeq, homology and de-novo gene predictions to comprehensively annotate the set of coding genes and infer a phylogeny with estimated species divergence times. This genome and its annotation is certainly a valuable resource for future studies.

Major comments:
1) The Busco scores indicate that this assembly has fewer complete but more fragmented genes, compared to P. alecto and R. aegyptiacus. Given the high contig N50 value, this is unexpected. Therefore I wonder whether this result is caused by remaining sequencing errors in the assembly, even after 80X PacBio data. I would suggest to test this by asking how many of the genes with clear homology to other bats or mammals have a complete open reading frame (ATG .. stop codon) without a frameshift, and how many genes have frameshifts that could account for the higher number of partial transcripts. I also wonder if any Illumina data (genomic or RNA-seq) is available to potentially correct such errors.

2) "Our results also indicated that the complexity of the bat transcriptome is similar to that of the human" While this may be correct, this conclusion is not justified by the data. A comparable, much deeper transcriptome profiling would be necessary to investigate this. I would remove this sentence.

Minor comments:
1) "we found that known REs accounted for 34.65% in Espe.v1, which is the highest proportion amongst all bat genomes published to date"
Correct, but it should be mentioned that the difference to R. aegyptiacus is just 0.02%.

2) Why was D. melanogaster used as an outgroup to identify single copy mammalian genes and not a more closely-related species?

3) Related to this point, 'only' 1212 single copy genes appears to be a very low number. While this is likely sufficient for phylogenetic inference, I would have expected >10000 such genes. Maybe this is caused by using D. mel as the outgroup where fewer genes will align to?

4) As a comment: The higher isoform to gene ratio for human is expected as human has much deeper transcriptome resources of many more tissues.

5) "de novo assembly of a non-reference, non-model genome" --> I believe 'non-reference' is redundant and can be removed as the sentence already has 'de novo assembly'

6) typo
Proteinortho software (v. 5.16b) [27] was used to identified
--> Proteinortho software (v. 5.16b) [27] was used to identify

Level of Interest

Please indicate how interesting you found the manuscript: Choose an item.

Quality of Written English

Please indicate the quality of language in the manuscript: Choose an item.

Declaration of Competing Interests

Please complete a declaration of competing interests, considering the following questions:

- Have you in the past five years received reimbursements, fees, funding, or salary from an organisation that may in any way gain or lose financially from the publication of this manuscript, either now or in the future?
- Do you hold any stocks or shares in an organisation that may in any way gain or lose financially from the publication of this manuscript, either now or in the future?
- Do you hold or are you currently applying for any patents relating to the content of the manuscript?
- Have you received reimbursements, fees, funding, or salary from an organization that holds or has applied for patents relating to the content of the manuscript?
- Do you have any other financial competing interests?
- Do you have any non-financial competing interests in relation to this paper?

If you can answer no to all of the above, write 'I declare that I have no competing interests' below. If your reply is yes to any, please give details below.

None.

I agree to the open peer review policy of the journal. I understand that my name will be included on my report to the authors and, if the manuscript is accepted for publication, my named report including any attachments I upload will be posted on the website along with the authors' responses. I agree for my report to be made available under an Open Access Creative Commons CC-BY license (http://creativecommons.org/licenses/by/4.0/). I understand that any comments which I do not wish to be included in my named report can be included as confidential comments to the editors, which will not be published.

I agree to the open peer review policy of the journal

To further support our reviewers, we have joined with Publons, where you can gain additional credit to further highlight your hard work (see: https://publons.com/journal/530/gigascience). On publication of this paper, your review will be automatically added to Publons, you can then choose whether or not to claim your Publons credit. I understand this statement. Yes
