# Supplementary material for: Exploring the genome and transcriptome of the cave nectar bat Eonycteris spelaea with PacBio long-read sequencing
Source: Gigascience. 2018 Sep 20;7(10):giy116. doi: 10.1093/gigascience/giy116 (PMC6177735; doi:10.1093/gigascience/giy116)
Supplement: Reviewer_2_Report_(Original_Submission) -- Xiuguang Mao — 4/24/2018 Reviewed [file giy116_reviewer_2_report_(original_submission).pdf]

Reviewer Report

Title: Exploring the genome and transcriptome of the cave nectar bat Eonycteris spelaea with PacBio long-read sequencing

Version: Original Submission **Date:** 4/24/2018

Reviewer name: Xiuguang Mao

Reviewer Comments to Author:

Comments:
One of the main aim of this MS is to investigate whether the PacBio long-read sequencing platform alone is sufficient to explore the whole-genome and transcriptome of an organism with a large genome size (~2Gb). The authors used the cave nectar bat as the study system possibly because of their long interests in studying immune and virus in megabats. I remember that the authors' lab was the first to explore the bat transcriptome using the second generation sequencing platform. This time, the authors' lab was the first to explore the bat genome and transcriptome using the PacBio sequencing platform alone.

However, reads generated by PacBio sequencing may contain high error rate. An alternative to using PacBio sequencing alone is to develop error correction strategies using hybrid sequencing, such as in the case of another megabat genome of Rousettus aegyptiacus which was sequenced using both short reads from the illumina HiSeq platform and long reads from the PacBio platform). The authors did little work to evaluate the error rate of PacBio sequencing in the study (e.g. by comparing with Sanger sequencing or illumina HiSeq). In addition, the authors may need to evaluate the strength and weakness of using PacBio sequencing alone and hybrid sequencing for eukaryotic de novo genome assemblies.

Since the close relationship between Rousettus aegyptiacus (whose genome was sequenced using hybrid sequencing platform) and Eonycteris spelaea, the authors should provide more backgrounds for readers to understand why they spend so much of money to sequence the genome of Eonycteris spelaea.

I suggest the authors to provide more details for the reasons why they did phylogenetic analysis in the study; currently I do not think that this analysis is quite necessary.

Detailed comments:
Line 71, Myotis rufoniger was not included in Table S1, and no assembled genome was reported for this species. So this species can be excluded here?
Line 113, "were extracted from two individuals" should be "was extracted from two individuals".
Line 114, "…bats were then pooled together" should be "…bats was then pooled together"
Line 115, "…insert sizes were generate…" should be "…insert sizes were generated…"
Line 131, "…were present in the assemble…" should be "…was present in the assemble…"
Line 190, "… PacBio® Iso-Seq technology have significantly…" should be "… PacBio® Iso-Seq technology has significantly…"
Line 234, "Tissues were homogenized and RNA extracted" should be "Tissues were homogenized and RNA was extracted"
Line 236, "Extracted RNA were subsequently eluted" should be "Extracted RNA was subsequently eluted"
Line 272, "Gene Annotation" should be "Genome Annotation" ?
Line 276, please add references for Augustus and SNAP
Line 287, "Maker predict transcript were merged…" should be "Maker predict transcripts were merged…"
Line 289-291, please restated this sentence.
Line 295, "was used to identified the…" should be "was used to identify the…"
Line 299, "…each single-copy family were aligned…" should be "…each single-copy family was aligned…"
Line 300, "…sites was extracted from each…" should be "…sites were extracted from each…"

Level of Interest

Please indicate how interesting you found the manuscript: Choose an item.

Quality of Written English

Please indicate the quality of language in the manuscript: Choose an item.

Declaration of Competing Interests

Please complete a declaration of competing interests, considering the following questions:

- Have you in the past five years received reimbursements, fees, funding, or salary from an organisation that may in any way gain or lose financially from the publication of this manuscript, either now or in the future?
- Do you hold any stocks or shares in an organisation that may in any way gain or lose financially from the publication of this manuscript, either now or in the future?
- Do you hold or are you currently applying for any patents relating to the content of the manuscript?
- Have you received reimbursements, fees, funding, or salary from an organization that holds or has applied for patents relating to the content of the manuscript?
- Do you have any other financial competing interests?
- Do you have any non-financial competing interests in relation to this paper?

If you can answer no to all of the above, write 'I declare that I have no competing interests' below. If your reply is yes to any, please give details below.

I declare that I have no competing interests

I agree to the open peer review policy of the journal. I understand that my name will be included on my report to the authors and, if the manuscript is accepted for publication, my named report including any attachments I upload will be posted on the website along with the authors' responses. I agree for my report to be made available under an Open Access Creative Commons CC-BY license (http://creativecommons.org/licenses/by/4.0/). I understand that any comments which I do not wish to be included in my named report can be included as confidential comments to the editors, which will not be published.

I agree to the open peer review policy of the journal

To further support our reviewers, we have joined with Publons, where you can gain additional credit to further highlight your hard work (see: https://publons.com/journal/530/gigascience). On publication of this paper, your review will be automatically added to Publons, you can then choose whether or not to claim your Publons credit. I understand this statement. Yes
