# Supplementary material for: Exploring the genome and transcriptome of the cave nectar bat Eonycteris spelaea with PacBio long-read sequencing
Source: Gigascience. 2018 Sep 20;7(10):giy116. doi: 10.1093/gigascience/giy116 (PMC6177735; doi:10.1093/gigascience/giy116)
Supplement: Reviewer_3_Report_(Original_Submission) -- Graham Hughes — 5/1/2018 Reviewed [file giy116_reviewer_3_report_(original_submission).pdf]

Reviewer Report

Title: Exploring the genome and transcriptome of the cave nectar bat Eonycteris spelaea with PacBio long-read sequencing

Version: Original Submission **Date:** 5/1/2018

Reviewer name: Graham Hughes

Reviewer Comments to Author:

Reviewer's comments
Major points
1) P.5 lines 101 - 107, 112 -119: Given the intense details of these sections, they read more like a specific set of materials and methods, and should be placed in the appropriate section
2) I am a little concerned about the usage of D. mealogaster as an out-group for the author's phylogenetic analyses. Their datasets used to analyze phylogeny contains 12 mammalian genomes and 1 arthropod. I am worried that this may affect divergence time estimates. Perhaps the authors should consider using vertebrate taxon as an out-group, or at least state their reasoning for using an invertebrate as an out-group for a mammalian phylogeny.
3) In Table 2, combining PacBio and Illumina reads results in less coding genes for R. aegyptiacus than either genome where only one method is utilized. This is despite the fact that there are very similar GC level and repetitive elements between E. spelaea and R. aegyptiacus, with both appearing as sister taxa in the described phylogeny. The authors should comment on, discuss, or explain such a stark difference (independent methods providing more coding genes than combined methods).
4) The authors have missed out on an opportunity here to comment on the utility of PacBio long reads only in genome assembly (E. spelaea) compared to a genome assembly with both PacBio and Illumina reads (R. aegyptiacus), as this may have implications for researchers trying to find the optimum, most cost affective means of genome assembly. A short comment in the discussion should be included discussing this.
Minor points
p. 2, line 29: Authors use "aging", but use "ageing" in line 66. Please use consistent spelling.
p. 3, line 57: Perhaps the statement "Unique amongst the mammalian species and as the only group of mammals with true powered flight" would be better written as "Unique amongst mammalian species due to being the only order with true powered flight"
p. 3, line 62: Sentence used plural 'comparing' but past tense 'revealed', authors shoud say "has revealed".
p.3 line 62-65: very long, sentence. Consider breaking up.
p.4 Line 83. This sentences suggests only 1 bat genome exists, consider "annotate the genomes of other bats" as an alternative.
p. 6 line 128: should it be "we employed the BUSCO (v.3) method" rather than "employed BUSCO method"?
p. 6 line 132: change gene sets identification to gene set identification
p. 7 line 143: Known REs accounted for 34.65% of what? The genome? Or of all repetitive elements detected?
p. 7 line 144: Should Espe.v1 get a citation?
p. 8, line 169: should be "closest sister taxon to R. aegyptiacus" rather than "closest to"
p. 11, line 243: What length were the sub-reads?
p. 11, Genome assembly section: Could the authors provide a short single sentence description of what each software does, in addition to providing the parameters, to give greater context.

Level of Interest

Please indicate how interesting you found the manuscript: Choose an item.

Quality of Written English

Please indicate the quality of language in the manuscript: Choose an item.

Declaration of Competing Interests

Please complete a declaration of competing interests, considering the following questions:

- Have you in the past five years received reimbursements, fees, funding, or salary from an organisation that may in any way gain or lose financially from the publication of this manuscript, either now or in the future?
- Do you hold any stocks or shares in an organisation that may in any way gain or lose financially from the publication of this manuscript, either now or in the future?
- Do you hold or are you currently applying for any patents relating to the content of the manuscript?
- Have you received reimbursements, fees, funding, or salary from an organization that holds or has applied for patents relating to the content of the manuscript?
- Do you have any other financial competing interests?
- Do you have any non-financial competing interests in relation to this paper?

If you can answer no to all of the above, write 'I declare that I have no competing interests' below. If your reply is yes to any, please give details below.

I declare that I have no competing interests

I agree to the open peer review policy of the journal. I understand that my name will be included on my report to the authors and, if the manuscript is accepted for publication, my named report including any attachments I upload will be posted on the website along with the authors' responses. I agree for my report to be made available under an Open Access Creative Commons CC-BY license (http://creativecommons.org/licenses/by/4.0/). I understand that any comments which I do not wish to be included in my named report can be included as confidential comments to the editors, which will not be published.

I agree to the open peer review policy of the journal

To further support our reviewers, we have joined with Publons, where you can gain additional credit to further highlight your hard work (see: https://publons.com/journal/530/gigascience). On publication of this paper, your review will be automatically added to Publons, you can then choose whether or not to claim your Publons credit. I understand this statement. Yes
