# Supplementary material for: Exploring the genome and transcriptome of the cave nectar bat Eonycteris spelaea with PacBio long-read sequencing
Source: Gigascience. 2018 Sep 20;7(10):giy116. doi: 10.1093/gigascience/giy116 (PMC6177735; doi:10.1093/gigascience/giy116)
Supplement: Reviewer_2_Report_Revision_1 -- Xiuguang Mao — 8/3/2018 Reviewed [file giy116_reviewer_2_report_revision_1.pdf]

Reviewer Report

Title: Exploring the genome and transcriptome of the cave nectar bat Eonycteris spelaea with PacBio long-read sequencing

Version: Revision 1 Date: 8/3/2018

Reviewer name: Xiuguang Mao

Reviewer Comments to Author:

Major comments:
When compared to the hybrid assembled R. aegyptiacus, the authors stated "combined, there are 0.7% more fragmented or missing genes than that of R. aegyptiacus in the BUSCO report". I cannot follow their explanation in the following sentence "This slight decrease in gene integrity……". Should be "the slight increase in gene integrity of R. aegyptiacus……"? please clarify this.

Detailed comments:
Line 153, "18,588 (90.1%) of the coding genes were supported by at least two types of prediction evidences……" should be "18,588 (90.1%) of the coding genes were supported by at least one of the two types of prediction evidences……"?
Line 167, "……is consistent with previous studies" should be "……are consistent with previous studies". And please add references for previous studies.
Line 227, "therefore lowly expressed transcripts could have been missed due to the lack of depth" can be deleted here?
Line 231, "…… becomes ……" should be "… become ……"
Line 286, "…build…" should be "…built…"
Line 289, "… Primary…" should be "… primary…"
Line 334, "…orthologous gene…" should be "…orthologous genes…"
Line 337, "… gene alignments …" should be "… gene alignment …"

Level of Interest

Please indicate how interesting you found the manuscript: Choose an item.

Quality of Written English

Please indicate the quality of language in the manuscript: Choose an item.

Declaration of Competing Interests

Please complete a declaration of competing interests, considering the following questions:

- Have you in the past five years received reimbursements, fees, funding, or salary from an organisation that may in any way gain or lose financially from the publication of this manuscript, either now or in the future?
- Do you hold any stocks or shares in an organisation that may in any way gain or lose financially from the publication of this manuscript, either now or in the future?

- Do you hold or are you currently applying for any patents relating to the content of the manuscript?
- Have you received reimbursements, fees, funding, or salary from an organization that holds or has applied for patents relating to the content of the manuscript?
- Do you have any other financial competing interests?
- Do you have any non-financial competing interests in relation to this paper?

If you can answer no to all of the above, write 'I declare that I have no competing interests' below. If your reply is yes to any, please give details below.

I declare that I have no competing interests

I agree to the open peer review policy of the journal. I understand that my name will be included on my report to the authors and, if the manuscript is accepted for publication, my named report including any attachments I upload will be posted on the website along with the authors' responses. I agree for my report to be made available under an Open Access Creative Commons CC-BY license (http://creativecommons.org/licenses/by/4.0/). I understand that any comments which I do not wish to be included in my named report can be included as confidential comments to the editors, which will not be published.

I agree to the open peer review policy of the journal

To further support our reviewers, we have joined with Publons, where you can gain additional credit to further highlight your hard work (see: https://publons.com/journal/530/gigascience). On publication of this paper, your review will be automatically added to Publons, you can then choose whether or not to claim your Publons credit. I understand this statement. Yes
